# Re-randomisation trials in multi-episode settings: Estimands and independence estimators

**DOI:** 10.1177/09622802221094140

**Published:** 2022-04-14

**Authors:** Brennan C Kahan, Ian R White, Richard Hooper, Sandra Eldridge

**Affiliations:** 1 Pragmatic Clinical Trials Unit, 4617 Queen Mary University of London, London, UK; 2 524254 MRC Clinical Trials Unit at UCL, London, UK

**Keywords:** Estimand, re-randomisation design, randomised trial, multi-episode setting, informative cluster size

## Abstract

Often patients may require treatment on multiple occasions. The re-randomisation design can be used in such multi-episode settings, as it allows patients to be re-enrolled and re-randomised for each new treatment episode they experience. We propose a set of estimands that can be used in multi-episode settings, focusing on issues unique to multi-episode settings, namely how each episode should be weighted, how the patient's treatment history in previous episodes should be handled, and whether episode-specific effects or average effects across all episodes should be used. We then propose independence estimators for each estimand, and show the manner in which many re-randomisation trials have been analysed in the past (a simple comparison between all intervention episodes vs. all control episodes) corresponds to a per-episode added-benefit estimand, that is, the average effect of the intervention across all episodes, over and above any benefit conferred from the intervention in previous episodes. We show this estimator is generally unbiased, and describe when other estimators will be unbiased. We conclude that (i) consideration of these estimands can help guide the choice of which analysis method is most appropriate; and (ii) the re-randomisation design with an independence estimator can be a useful approach in multi-episode settings.

## 1 Introduction

In many clinical settings, patients may require treatment on multiple occasions. For example, patients who experience acute sickle cell pain crises will require treatment for each pain crisis they experience; patients who experience severe asthma exacerbations will require treatment for each new exacerbation; and patients who develop febrile neutropenia as a result of chemotherapy would require treatment for each new round of chemotherapy leading to a neutropenic episode.^1–3^ In these settings, patients would typically be given the same treatment for each new episode. We refer to these as ‘multi-episode’ settings.

Re-randomisation trials have been used to evaluate interventions in multi-episode settings.^4–6^ The re-randomisation design involves re-enrolling and re-randomising patients for each new episode they experience; overviews of this design are available in references.^1,4,5,7^ Importantly, the number of times each patient is enrolled in the trial is not specified in advance, but depends on how many treatment episodes they experience during the trial.^5^

The re-randomisation design has been applied to trials of acute sickle cell pain crises,^2^ severe asthma exacerbations,^3^ influenza vaccination,^8^ platelet transfusions for thrombocytopenia,^9^ complications of cirrhosis,^10^ febrile neutropenia,^11^ ambient light to perform biophysical profiles,^12^ pre-term births,^13^ and in-vitro fertilisation.^14^ These trials are typically analysed by comparing all episodes allocated to the intervention versus all episodes allocated to the control, for instance using a *t*-test or linear regression model on the individual episodes^1^ (counterintuitively, the approach of ignoring within-patient correlation between episodes can provide valid standard errors under certain assumptions, e.g. see Dunning and Reeves^4^ and Kahan et al.,^5^ although robust standard errors which allow for clustering may be more appropriate in case these assumptions fail^15^).

With the publication of the ICH-E9 addendum,^16^ there is growing recognition of the importance of estimands^17–22^ (a precise definition of the treatment effect to be estimated), as careful consideration of the estimand can clarify research objectives and ensure the trial design and statistical methods are aligned with those objectives. However, estimands have not yet been defined for the re-randomisation design, or for multi-episode settings more generally. Therefore, it is not immediately clear what estimand the analysis approach discussed above corresponds to, or whether this estimand is in line with trial objectives.

In this article, we (i) propose a set of estimands that can be used for multi-episode settings; (ii) show that the analysis approach used in previous re-randomisation trials corresponds to one of these proposed estimands (the ‘per-episode added-benefit’ estimand); (iii) propose a set of estimators for the re-randomisation design which correspond to the proposed estimands; (iv) discuss the bias of these estimators; and (v) discuss these estimands in the context of a trial of sickle cell pain crises. We focus on independence estimators (where estimates are obtained using a working independence correlation structure, often in conjunction with robust standard errors). For simplicity, we limit ourselves to the setting where the interventions under study do not affect whether future episodes occur; that is, patients would experience the same number of episodes during the trial period regardless of which treatments they receive. We note that re-randomisation trials could still be used if treatment affects the occurrence of future episodes (e.g. see Nason and Follmann^6^), but that some of the estimands specified in this article would not be directly applicable. However, we do allow for non-enrolment in this article (where future episodes do occur, but patients are not re-enrolled into the trial).

## 2 Notation

Let *i* index patient, and *j* index the episode number within the trial. $M_{i}$ represents the number of episodes for which patient *i* is enrolled in the trial, $M_{T}$ the total number of episodes enrolled, and $M_{T(j)}$ the total number of patients for whom $M_{i}=j$ (i.e. the number of patients enrolled for *j* episodes). There are $N_{T}$ total patients enrolled in the trial, and $N_{j}$ represents the number of patients who are enrolled for at least *j* episodes. For example, $M_{i}=3$ indicates patient *i* was enrolled for three episodes, and $N_{3}=45$ denotes there were 45 patients enrolled in the trial at episode 3.

Let $Y_{ij}$ denote the outcome for patient *i* during episode *j*, and $Z_{ij}$ denote the treatment allocation (where 0 = control, 1 = intervention). ${\tilde{Z}}_{ij}$ represents the treatment allocations in the patient's previous episodes (referred to as the ‘treatment history’). For example, ${\tilde{Z}}_{13}$ would be the vector $\left(Z_{11},Z_{12}\right)$ .

Let $Y_{ij}^{\left(Z_{ij}=0,{\tilde{Z}}_{ij}={\tilde{z}}_{ij}\right)}$ represent patient i 's potential outcome at episode *j* under Z=0 and treatment history ${\tilde{Z}}_{ij}={\tilde{z}}_{ij}$ (and similarly for Z = 1). For clarity, we drop subscripts inside the brackets, as these are the same as subscripts on the outside of the brackets; for example, $Y_{ij}^{\left(Z=1,\tilde{Z}=\tilde{z}\right)}$ is the same as $Y_{ij}^{\left(Z_{ij}=1,{\tilde{Z}}_{ij}={\tilde{z}}_{ij}\right)}$ .

## 3 Estimands

An estimand ‘summarises at a population level what the outcomes would be in the same patients under different treatment conditions being compared’.^16^ It consists of five components: (1) the treatment regimens; (2) the population; (3) the outcome; (4) a population-level summary denoting how outcomes between treatment arms will be compared; and (5) how intercurrent events which may influence the interpretation of the treatment effect, such as treatment discontinuation, will be handled.

All of these components will need to be defined for re-randomisation trials. For some components, the considerations will generally be the same in a re-randomisation trial as in a parallel group or alternate trial design (e.g. the outcome definition, or how treatment discontinuation is handled), and so we do not discuss those here; instead, we focus on the aspects that are unique to multi-episode settings, and thus to re-randomisation trials.

The three aspects we focus on in this article are (i) whether each episode or each patient is given equal weight in the estimand; (ii) how the patient's ‘treatment history’ (i.e. the treatments they were allocated to in previous episodes) is handled; and (iii) the set of episodes included in the estimand.

### 3.1 Weighting by episode or by patient: Per-episode versus per-patient estimands

Here we discuss whether to give equal weight to each episode or to each patient in calculating the treatment effect.

Consider a setting where patients with more severe underlying diseases are pre-disposed to experience a larger number of episodes, but are less likely to respond to intervention than other patients. Imagine the treatment effect is $\beta_{1}$ for patients who experience one episode, $\beta_{2}$ for patients who experience two episodes, and that 50% of patients experience one episode and 50% experience two episodes. Then, the average treatment effect if we give equal weight to each patient is $0.5\beta_{1}+0.5\beta_{2}$ , whereas the average treatment effect if we give equal weight to each episode is $0.33\beta_{1}+0.67\beta_{2}$ (this difference comes from weighting the treatment effects by the percentage of patients vs. by the percentage of episodes that they correspond to).

In this section, we define two different approaches to weighting: the per-episode estimand, and the per-patient estimand. The approach we use to define these estimands is based on a sampling scheme framework, as has been used to define estimands in other settings (notably in the informative cluster size setting).^23–29^

#### 3.1.1 Per-episode estimands

The per-episode estimand (denoted by $\beta_{E}$ ) gives the average effect across all episodes. For instance, in the example listed above, the per-episode estimand would weigh the treatment effects $\beta_{1}$ and $\beta_{2}$ according to the number of episodes, they correspond to.

Let *I* and *J* be random variables, where I=i and J=j with a specified probability; *I* represents a randomly selected patient from the trial, and *J* represents a randomly selected episode from patient I=i . Then, $Y_{IJ}$ represents the outcome for a randomly selected episode from the trial, where each episode has a certain probability of being selected.

Then, let $Y_{{\left(IJ\right)}^{E}}$ denote an episode selected completely at random (i.e. where each episode has an equal probability of being selected). This is accomplished by letting $Y_{{\left(IJ\right)}^{E}}$ denote $Y_{I^{E}J^{E}}$ where $I^{E}=i$ with probability $\frac{M_{i}}{M_{T}}$ , and, conditional on $I^{E}$ , $J^{E}$ is uniformly distributed on $\left(1,\ldots ,M_{I^{E}}\right)$ (where $M_{I^{E}}$ represents the total number of episodes, $M_{i}$ , for which patient $I^{E}$ was enrolled in the trial). By multiplying these two probabilities $\left(\frac{M_{i}}{M_{T}}\mathrm{and}\frac{1}{M_{i}}\right)$ , $Y_{{\left(IJ\right)}^{E}}$ represents the outcome from a randomly selected episode from the trial, where each episode is selected with equal probability, equivalent to $\frac{1}{M_{T}}$ .

Then, the estimand is defined as

$$\beta_{E}=E(Y_{{\left(IJ\right)}^{E}}^{\left(Z=1\right)}-Y_{{\left(IJ\right)}^{E}}^{\left(Z=0\right)})$$

That is, it is the difference in potential outcomes, averaged across all episodes. This estimand can be related to the potential outcomes by the expression

$$\beta_{E}=\frac{1}{M_{T}}\underset{ij}{\sum }\;\beta_{ij}$$

where $\beta_{ij}=Y_{ij}^{\left(Z=1\right)}-Y_{ij}^{\left(Z=0\right)}$ (i.e. the potential treatment effect for patient *i* at episode j ).

#### 3.1.2 Per-patient estimands

The per-patient estimand (denoted by $\beta_{P}$ ) gives the average effect across patients. Consider again the example listed earlier; the per-patient estimand would weigh the treatment effects $\beta_{1}$ and $\beta_{2}$ according to the number of patients they correspond to (rather than according to the number of episodes, as in the per-episode estimand).

Let $Y_{{\left(IJ\right)}^{P}}$ denote $Y_{I^{P}J^{P}}$ where $I^{P}$ has a uniform distribution on $\left(1,\ldots ,N_{T}\right)$ , and, conditional on $I^{P}$ , $J^{P}$ is uniformly distributed on $\left(1,\ldots ,M_{I^{P}}\right)$ (where $M_{I^{P}}$ represents the value of $M_{i}$ for the randomly selected patient $I^{P}$ ). Then $Y_{{\left(IJ\right)}^{P}}$ denotes the outcome from a randomly selected episode from a randomly selected patient. It is based on a two-stage sampling scheme, where a patient is randomly selected in the first stage (each with equal probability), then an episode from within that patient is randomly selected in the second stage (each with equal probability). Because there are $N_{T}$ patients in the population of interest, the probability of selection for each patient is $\frac{1}{N_{T}}$ , and because each patient has $M_{i}$ episodes, the probability of selection for each episode, given that patient *i* has been selected, is $\frac{1}{M_{i}}$ . Therefore, the overall probability of selection for each episode is $\frac{1}{N_{T}}\frac{1}{M_{i}}$ It can be seen as taking an average of the patient-specific treatment effects (where each patient-specific treatment effect is the average treatment effect for that patient over the episodes they experience).

The estimand is defined as

$$\beta_{P}=E(Y_{{\left(IJ\right)}^{P}}^{\left(Z=1\right)}-Y_{{\left(IJ\right)}^{P}}^{\left(Z=0\right)})$$

This estimand can be related to the potential outcomes by the expression

$$\beta_{P}=\frac{1}{N_{T}}\underset{ij}{\sum }\frac{1}{M_{i}}\beta_{ij}$$

### 3.2 Treatment history: Added-benefit versus policy-benefit estimands

One key difference in the multi-episode setting compared to many other settings with clustered data is that in the multi-episode setting episodes occur sequentially in time, and outcomes or treatment effects in a patient's current episode may depend on the treatments they received in previous episodes. For example, imagine that patient outcomes follow the model

(1) $$Y_{ij}=\alpha +\beta Z_{ij}+\gamma Z_{i,j-1}+\mu_{i}+\varepsilon_{ij}$$

where $Z_{i,j-1}$ represents the treatment in a patient's previous episode (and is defined as 0 for the patient's first episode). Under this model, the intervention effect carries forward into the next episode by the amount γ . Conditional on the treatment received in episode one, the treatment effect in episode two is β . However, we may wish to assess the effect of a treatment policy, where patients receive the intervention for all episodes versus the control for all episodes (i.e. at episode two it would compare treatment sequences Z=(1,1) vs. Z=(0,0) ). In this case, the treatment effect at episode two would be β+γ . We can therefore define different estimands based on different ways of incorporating a patient's treatment history.

In this section, we define two different approaches for incorporating treatment history into the estimand definition: the policy-benefit estimand, and the added-benefit estimand. For the moment, we do not distinguish between per-episode and per-patient sampling schemes, and instead, use $Y_{IJ}$ as a placeholder for one of these two sampling schemes.

#### 3.2.1 Policy-benefit estimands

The policy-benefit estimand (denoted by $\beta^{PB}$ ) gives the effect of a treatment policy where patients are allocated to either receive intervention for all episodes, or control for all episodes.

We define the policy-benefit estimand as the expected difference in potential outcomes for a randomly selected episode (based either on a per-episode or per-patient basis), which has been allocated intervention for the current and all previous episodes versus control for the current and all previous episodes. It can be thought of as the average effect of always versus never treating.

The estimand can be written as

$$\beta^{PB}=E(Y_{IJ}^{\left(Z=1,\tilde{Z}=\tilde{1}\right)}-Y_{IJ}^{\left(Z=0,\tilde{Z}=\tilde{0}\right)})$$

where $\tilde{Z}=\tilde{1}$ means the patient was assigned to intervention for all previous episodes in the trial, and $\tilde{Z}=\tilde{0}$ means they were assigned to control for all previous episodes.

This estimand can be related to the potential outcomes by the expression

$$\beta^{PB}=\frac{1}{M_{T}}\underset{ij}{\sum }\;\beta_{ij}^{PB}$$

where $\beta_{ij}^{PB}=Y_{ij}^{\left(Z=1,\tilde{Z}=\tilde{1}\right)}-Y_{ij}^{\left(Z=0,\tilde{Z}=0\right)}$ (i.e. it represents the difference in potential outcomes for patient *i* at episode *j* under a policy of all intervention vs. all control). (The above expression relates to a per-episode approach, but could easily be adapted to a per-patient strategy.)

#### 3.2.2 Added-benefit estimands

The added-benefit estimand (denoted by $\beta^{AB}$ ) gives the additional effect of being assigned the intervention in the current episode (i.e. ‘what is the benefit of the intervention in this episode, over and above the benefit from previous episodes?’). Consider again model (1); the added-benefit effect would omit the term γ , as this represents carried-over benefit from previous episodes, rather than any new benefit from the intervention in the current episode.

We define the added-benefit estimand as the expected difference in potential outcomes for a randomly selected episode (based either on a per-episode or per-patient basis), based on both the intervention and control potential outcomes sharing the same treatment history.

The estimand can be written as

$$\beta^{AB}=E(Y_{IJ}^{\left(Z=1,\tilde{Z}\right)}-Y_{IJ}^{\left(Z=0,\tilde{Z}\right)})$$

Here, the expectation is over both the IJ and the distribution of $\tilde{Z}$ , that is, the expectation is taken over the different treatment histories according to their probability of being observed for each patient. The reason for this is that the treatment effect may depend on treatment history; for instance, if the intervention became more or less effective the more often it is used, then its benefit in a particular episode will depend on the number of times a patient has received it previously. This estimand represents a weighted average across all possible treatment histories, with weights based on the probability of treatment history $\tilde{Z}=\tilde{z}$ being observed at episode *j* for patient *i*. This probability, denoted as $P({\tilde{Z}}_{ij}={\tilde{z}}_{ij})$ , depends on two factors; the allocation probabilities used in the study (e.g. 1:1 allocation ratio vs. 2:1 allocation ratio), and whether the patient would be enrolled in the trial at episode *j* under treatment history ${\tilde{Z}}_{ij}={\tilde{z}}_{ij}$ (for instance, patients who experience two episodes may decide to enrol in the trial for their second episode under one treatment history, but not under a different history; if they would not enrol for a certain episode, that episode is not included in the estimand). Note that this probability is not known in practice, as although we know the allocation ratio, we will not know whether patients would be enrolled in the trial under different treatment histories.

The estimand can be related to the potential outcomes as follows

$$\beta^{AB}=\frac{1}{M_{T}}\underset{ij}{\sum }\;{\bar{\beta }}_{ij}^{AB}$$

where $\beta_{ij}^{AB(\tilde{Z}=\tilde{z})}=Y_{ij}^{\left(Z=1,\tilde{Z}=\tilde{z}\right)}-Y_{ij}^{\left(Z=0,\tilde{Z}=\tilde{z}\right)}$ represent the difference in potential outcomes for patient *i* at episode *j* under treatment history $\tilde{Z}=\tilde{z}$ ; this can be thought of as the potential treatment effect for patient *i* at episode *j* under that specific treatment history. Then, ${\bar{\beta }}_{ij}^{AB}$ is a weighted average of the $\beta_{ij}^{AB(\tilde{Z}=\tilde{z})}$ , with weights equal to the probability of that treatment history being observed

$${\bar{\beta }}_{ij}^{AB}=\underset{{\tilde{Z}}_{ij}}{\sum }\;\beta_{ij}^{AB(\tilde{Z}=\tilde{z})}P({\tilde{Z}}_{ij}={\tilde{z}}_{ij})$$

where the summation $\underset{{\tilde{Z}}_{ij}}{\sum }\;(\cdot )$ is taken across all possible values of ${\tilde{Z}}_{ij}$ at episode *j*.

There are several important implications that follow on from this definition. The first is that the estimand depends on the distribution of $\tilde{Z}$ , and so changes to this distribution may lead to different values of the estimand (e.g. changing the allocation ratio from 1:1 to 2:1 will change the distribution of the treatment history, and hence the value of the estimand). This implication is inherent to any definition of the estimand which averages over different treatment histories. Of note, if the treatment effect is not affected by treatment history, then the true value of the estimand does not depend on the distribution of $\tilde{Z}$ . Then, the manner in which the treatment histories are weighted does not matter, as all definitions will be equivalent.

Another implication of this definition is that the estimand excludes episodes corresponding to treatment histories for which the patient would not be enrolled in the trial. For instance, if a patient would re-enrol in the trial for their second episode if they received intervention in the first episode, but not if they received control, then $P({\tilde{Z}}_{i2}=(1))=1$ and $P({\tilde{Z}}_{i2}=(0))=0$ , and hence $\beta_{i2}^{AB(\tilde{Z}=(1))}$ gets all the weight, and $\beta_{i2}^{AB(\tilde{Z}=(0))}$ is excluded from the calculation. One benefit of defining the estimand to exclude episodes that would not be enrolled in the trial is that this definition is compatible with the setting where treatment allocation may influence the occurrence of subsequent episodes (for instance, in a trial evaluating an intervention to induce pregnancy in couples with difficulty conceiving), as this definition excludes episodes which would not have occurred under specific treatment histories. Although we do not consider this setting in this paper, we feel it is useful for definitions to apply to more complicated settings when possible.

### 3.3 The set of episodes to include: Average effects across all episodes versus episode-specific estimands

In the previous sections, we defined estimands that included all enrolled episodes. These provide a single overall average treatment effect across all episodes (with the type of average being defined by whether a per-episode or per-patient approach is used). This is in line with common practice in randomised trials, where a single average effect is typically provided for the main estimand, recognising that this average effect will not necessarily correspond to the true effect within each specific subgroup.

In multi-episode settings, it may sometimes be useful to define episode-specific estimands (denoted by $\beta_{j}$ for episode j ). These estimands represent the treatment effect at episode *j* (note that $\beta_{j}$ only includes patients for whom $M_{i}\geq j$ ). For example, $\beta_{2}$ represents the treatment effect at episode 2, in patients with $M_{i}\geq 2$ . Episode-specific estimands can be defined under either an added-benefit or policy-benefit framework; however, we note the per-episode versus per-patient framework does not apply, as each patient with $M_{i}\geq j$ contributes only one episode for each $\beta_{j}$ .

Under the added-benefit framework, the episode-specific estimand is

$$\beta_{j}^{AB}=E(Y_{I^{ES}j}^{\left(Z=1,\tilde{Z}\right)}-Y_{I^{ES}j}^{\left(Z=0,\tilde{Z}\right)})$$

And under the policy-benefit framework, the estimand can be defined as

$$\beta_{j}^{PB}=E(Y_{I^{ES}j}^{\left(Z=1,\tilde{Z}=\tilde{1}\right)}-Y_{I^{ES}j}^{\left(Z=0,\tilde{Z}=\tilde{0}\right)})$$

where $I^{ES}$ is a random variable which represents a randomly selected patient (with equal probability) at episode *j* (from the subset of patients with $M_{i}\geq j$ ). We use the ES superscript to denote episode-specific.

Of note, if we want to know whether the intervention is more effective the first time it is used versus the second time, a comparison between $\beta_{1}$ versus $\beta_{2}$ does not tell us this, because $\beta_{1}$ applies to patients for whom $M_{i}=1$ , whereas $\beta_{2}$ does not. If the treatment effect is different in those for whom $M_{i}=1$ compared to $M_{i}>1$ , then $\beta_{1}$ and $\beta_{2}$ may differ, even if the effect is the same both the first and second time the intervention is used.

An alternate way to define episode-specific estimands is to restrict the subset of patients for each $\beta_{j}$ to those where $M_{i}\geq c$ , where *c* is the number of episodes we are interested in. For example, if we want to know whether the treatment effect is the same the first three times the intervention is used, then we could restrict to the subset of patients for whom $M_{i}\geq 3$ and compare $\beta_{1(M_{i}\geq 3)}$ versus $\beta_{2(M_{i}\geq 3)}$ versus $\beta_{3(M_{i}\geq 3)}$ .

### 3.4 Comparison between estimands

A full set of the estimands described is shown in Table 1. We now discuss some of the differences between the per-episode versus per-patient, and the added- versus policy-benefit estimands.

**Table 1.** Summary of estimands for multi-episode settings.

Estimand	Definition	Relating estimand to potential outcomes	Description
Per-episode added-benefit	$\beta_{E}^{AB}=E(Y_{{\left(IJ\right)}^{E}}^{\left(Z=1,\tilde{Z}\right)}-Y_{{\left(IJ\right)}^{E}}^{\left(Z=0,\tilde{Z}\right)})$	$\beta_{E}^{AB}=\frac{1}{M_{T}}\underset{ij}{\sum }\;{\bar{\beta }}_{ij}^{AB}$	Provides the additional effect of being assigned the intervention in the current episode, over and above the benefit of being assigned the intervention in previous episodes Provides an average effect across episodes
Per-episode policy-benefit	$\beta_{E}^{PB}=E(Y_{{\left(IJ\right)}^{E}}^{\left(Z=1,\tilde{Z}=\tilde{1}\right)}-Y_{{\left(IJ\right)}^{E}}^{\left(Z=0,\tilde{Z}=\tilde{0}\right)})$	$\beta_{E}^{PB}=\frac{1}{M_{T}}\underset{ij}{\sum }\;\beta_{ij}^{PB}$	Provides the effect of a treatment policy where patients are assigned intervention versus control for all episodes Provides an average effect across episodes
Per-patient added-benefit	$\beta_{P}^{AB}=E(Y_{{\left(IJ\right)}^{P}}^{\left(Z=1,\tilde{Z}\right)}-Y_{{\left(IJ\right)}^{P}}^{\left(Z=0,\tilde{Z}\right)})$	$\beta_{P}^{AB}=\frac{1}{N_{T}}\underset{ij}{\sum }\;\frac{1}{M_{i}}{\bar{\beta }}_{ij}^{AB}$	Provides the additional effect of being assigned the intervention in the current episode, over and above the benefit of being assigned the intervention in previous episodes Provides an average effect across patients
Per-patient policy-benefit	$\beta_{P}^{PB}=E(Y_{{\left(IJ\right)}^{P}}^{\left(Z=1,\tilde{Z}=\tilde{1}\right)}-Y_{{\left(IJ\right)}^{P}}^{\left(Z=0,\tilde{Z}=\tilde{0}\right)})$	$\beta_{P}^{PB}=\frac{1}{N_{T}}\underset{ij}{\sum }\;\frac{1}{M_{i}}\beta_{ij}^{PB}$	Provides the effect of a treatment policy where patients are assigned intervention versus control for all episodes Provides an average effect across patients
Episode-specific added-benefit	$\beta_{j}^{AB}=E(Y_{I^{ES}j}^{\left(Z=1,\tilde{Z}\right)}-Y_{I^{ES}j}^{\left(Z=0,\tilde{Z}\right)})$	$\beta_{j}^{AB}=\frac{1}{N_{j}}\underset{i:M_{i}\geq j}{\sum }\;{\bar{\beta }}_{ij}^{AB}$	Provides the additional effect of being assigned the intervention in the current episode, over and above the benefit of being assigned the intervention in previous episodes Provides an average effect at episode j , in patients with $M_{i}\geq j$
Episode-specific policy-benefit	$\beta_{j}^{PB}=E(Y_{I^{ES}j}^{\left(Z=1,\tilde{Z}=\tilde{1}\right)}-Y_{I^{ES}j}^{\left(Z=0,\tilde{Z}=\tilde{0}\right)})$	$\beta_{j}^{PB}=\frac{1}{N_{j}}\underset{i:M_{i}\geq j}{\sum }\;\beta_{ij}^{PB}$	Provides the effect of a treatment policy where patients are assigned intervention versus control for all episodes Provides an average effect at episode *j*, in patients with $M_{i}\geq j$

#### 3.4.1 Comparison between per-episode versus per-patient estimands

Whether the per-episode and per-patient estimands coincide will depend on whether there is ‘informative cluster size’,^23–29^ where the patient acts as the cluster, and the size is determined by $M_{i}$ . There are two types of informative cluster size: the first is when values of $Y_{ij}$ differ across different values of $M_{i}$ (e.g. patients who experience more episodes have different outcomes than those who experience fewer). The second is when the association between $Z_{ij}$ and $Y_{ij}$ is different across different values of $M_{i}$ , that is, the treatment effect in a given episode depends on the number of episodes for which that patient is enrolled.

For collapsible treatment effect measures (such as a difference in means, risk difference, or risk ratio), the per-episode and per-patient estimands should coincide unless the second type of informative cluster size occurs (where the treatment effect depends on $M_{i}$ ). In this case, the value of the two estimands will differ.

For non-collapsible effect measures (such as an odds ratio), the per-episode and per-patient will coincide in the absence of both types of informative cluster size; if either type occurs (either the outcome or treatment effect depends on $M_{i}$ ) the value of the two estimands will differ.

A simple way to evaluate whether the cluster size is informative under a particular data generating model (for a difference in means) is to take the mean of the potential outcomes and the mean of the potential treatment effects (either ${\bar{\beta }}_{ij}^{AB}$ or $\beta_{ij}^{PB}$ ) across episodes for each patient; then if the mean potential outcome for each patient differs according to $M_{i}$ , then the first type of informative cluster size has occurred, and if the mean potential treatment effect for each patient differs according to $M_{i}$ , then the second type has occurred (as this indicates the association between $Z_{ij}$ and $Y_{ij}$ is different across different values of $M_{i}$ ).

We provide a number of examples in the supplementary material of scenarios where these estimands either coincide or are different (for a difference in means).

We note that identifying informative cluster size can be more challenging for non-collapsible treatment effect measures, such as the odds ratio (OR). For instance, consider the setting where patients for whom $M_{i}=1$ and $M_{i}=2$ both have the same OR, but the baseline probability of an event differs between the two sets of patients. Here, the per-episode and per-patient estimands will provide the same conditional OR (conditional on $M_{i}$ ), but not the same marginal OR. Similarly, when considering covariate adjustment for baseline characteristics, the per-episode and per-patient conditional ORs will coincide provided the conditional OR does not vary across values of $M_{i}$ . Conversely, the marginal ORs will coincide provided the marginal OR does not vary across values of $M_{i}$ (i.e. the marginal OR based on all episodes for patients with a given value of $M_{i}$ ), and the baseline event rates do not vary by values of $M_{i}$ .

#### 3.4.2 Comparison between added-benefit versus policy-benefit estimands

The added-benefit and policy-benefit estimands will coincide if ${\bar{\beta }}_{ij}^{AB}=\beta_{ij}^{PB}$ . This will occur when the treatment history ${\tilde{Z}}_{ij}$ does not affect either $Y_{ij}^{\left(Z=0,\tilde{Z}=\tilde{z}\right)}$ or $Y_{ij}^{\left(Z=1,\tilde{Z}=\tilde{z}\right)}-Y_{ij}^{\left(Z=0,\tilde{Z}=\tilde{z}\right)}$ . When this is not the case then the added-benefit and policy-benefit estimands will usually differ.

We provide a number of examples in the supplementary material of scenarios where these estimands either coincide or are different.

## 4 Independence estimators

In this section, we describe a set of independence estimators that can be used in re-randomisation trials for the estimands described in Table 1. Independence estimators use a working independence correlation structure (i.e. they are based on a working assumption that episodes from the same patient are uncorrelated). We focus on a difference in means for a continuous outcome, however, the approaches in this section can be extended to estimate different types of treatment effects for different outcomes. They can be used in conjunction with robust standard errors which allow for clustering.^30^ We do not explicitly define estimators for the episode-specific estimands, though these can be easily adapted from the estimators listed.

Estimators are summarised in Table 2, along with an example Stata code to implement these estimators.

**Table 2.** Independence estimators.

Estimator	Formula	Stata code (version 15) to implement estimator
Per-episode added-benefit	${\hat{\beta }}_{E}^{AB}=\frac{\sum_{ij}\;Y_{ij}Z_{ij}}{\sum_{ij}\;Z_{ij}}-\frac{\sum_{ij}\;Y_{ij}(1-Z_{ij})}{\sum_{ij}\;(1-Z_{ij})}$	reg y z, vce(cluster id)
Per-patient added-benefit	${\hat{\beta }}_{P}^{AB}=\frac{\sum_{ij}\;W_{i}Y_{ij}Z_{ij}}{\sum_{ij}\;W_{i}Z_{ij}}-\frac{\sum_{ij}\;W_{i}Y_{ij}(1-Z_{ij})}{\sum_{ij}\;W_{i}(1-Z_{ij})}$	reg y z [pw = 1/m_i], vce(cluster id)
Per-episode policy-benefit	Step 1: $Y_{ij}=\alpha +\beta Z_{ij}+\gamma Z_{i,j-1}+\delta Z_{ij}Z_{i,j-1}+\beta_{ep}X_{ep_{ij}}+\varepsilon_{ij}$ Step 2: ${\hat{\beta }}_{E}^{PB}=\frac{N_{1}}{M_{T}}(\hat{\beta })+\frac{N_{2}}{M_{T}}(\hat{\gamma }+\hat{\beta }+\hat{\delta })$	reg y z##z_prev x_ep, vce(cluster id) lincom /// ‘prop_1st_ep’*_b[1.z] + /// ‘prop_2nd_ep’*(_b[1.z] + _b[1.z_prev] + _b[1.z#1.z\_prev])
Per-patient policy-benefit	Step 1: ${\hat{\beta }}_{P}^{PB}=\frac{1}{N_{T}}\underset{ij}{\sum }\;W_{i}{\hat{\beta }}_{ij}^{PB}$ Step 2: ${\hat{\beta }}_{P}^{PB}=\frac{M_{T(1)}}{N_{T}}(\hat{\beta })+\frac{M_{T(2)}}{N_{T}}\left(\frac{1}{2}\hat{\beta }+\left(\frac{1}{2}\right)(\hat{\beta }+\hat{\gamma }+\hat{\delta })\right)$	reg y z##z_prev x_ep [pw = 1/m_i], vce(cluster id) lincom /// ‘prop_has_1ep’*(_b[1.z]) + /// ‘prop_has_2ep’*((1/2)*(_b[1.z]) + /// (1/2)*(_b[1.z] + _b[1.z_prev] + _b[1.z#1.z_prev]))

Policy-benefit estimators are based on a maximum of two episodes per patient; this is because different causal models would need to be specified for the setting of ≥3 episodes. Added-benefit estimators can be used for any number of maximum episodes. For Stata code (version 15), ‘y’ denotes patient outcome, ‘z’ denotes treatment allocation, ‘id’ is a unique identifier for a patient, ‘m_i’ denotes the number of episodes for which the patient is enrolled in the trial, ‘z_prev’ denotes the patient's treatment allocation in their previous episode (and is set to 0 if it is the patient's first episode), ‘x_ep’ is an indicator for episode 2, ‘prop_1st_ep’ and ‘prop_2nd_ep’ represent the proportion of episodes in the trial which are first and second episodes, respectively, and ‘prop_has_1ep’ and ‘prop_has_2ep’ denote the proportion of patients enrolled in the trial for one and two episodes, respectively. In order to run the above code in Stata, ‘prop_1st_ep’, ‘prop_2nd_ep’, ‘prop_has_1ep’, and ‘prop_has_2ep’ must be saved as Stata local macros.

We provide some mathematical results evaluating the bias of the per-episode added-benefit and per-patient added-benefit estimators in the Supplemental Material and discuss when the policy-benefit estimators may be biased in the following sections.

### 4.1 Per-episode added-benefit estimator

The independence estimator for per-episode added-benefit estimand is

$${\hat{\beta }}_{E}^{AB}=\frac{\sum_{ij}\;Y_{ij}Z_{ij}}{\sum_{ij}\;Z_{ij}}-\frac{\sum_{ij}\;Y_{ij}(1-Z_{ij})}{\sum_{ij}\;(1-Z_{ij})}$$

That is, the per-episode added-benefit treatment effect is estimated as the difference in means between all intervention episodes versus all control episodes. This matches the analysis strategy that has typically been employed in previous re-randomisation trials,^1^ implying that analyses of these trials have targeted a per-episode added-benefit estimand.

In the Appendix, we show that this estimator is unbiased under a wide variety of different data-generating mechanisms.

### 4.2 Per-patient added-benefit estimator

The per-patient estimator can be obtained by weighting each patient by the inverse of their number of episodes, that is, $W_{i}=\frac{1}{M_{i}}$ :

$${\hat{\beta }}_{P}^{AB}=\frac{\sum_{ij}\;W_{i}Y_{ij}Z_{ij}}{\sum_{ij}\;W_{i}Z_{ij}}-\frac{\sum_{ij}\;W_{i}Y_{ij}(1-Z_{ij})}{\sum_{ij}\;W_{i}(1-Z_{ij})}$$

We show in the Appendix that the per-patient added-benefit estimator is unbiased under all data generating mechanisms we considered, except when there is differential non-enrolment. Non-enrolment occurs when a patient does not enrol in the trial for a particular episode; for instance, if they enrolled in the trial for the first episode they experience, but did not re-enrol for their second episode. We note that non-enrolment is not a form of dropout; patients only enrol in the trial for a single episode at a time, and there is no expectation that they must re-enrol for all subsequent episodes.

Differential non-enrolment occurs when different types of patients from the intervention and control groups re-enrol for their next episode. For example, in a setting where patients enrol for a maximum of two episodes, differential non-enrolment could occur if in the episode 1 intervention group, healthier patients were more likely to re-enrol in the trial for episode 2 than sicker patients, but in the episode 1 control group, sicker patients are more likely to re-enrol.

### 4.3 Per-episode policy-benefit estimator

To estimate the per-episode policy-benefit treatment effect, we must first estimate the $\beta_{ij}^{PB}$ 's (as defined earlier) and then use these to calculate the overall treatment effect. We can do this by specifying a causal model for the effect of treatment history ( ${\tilde{Z}}_{ij}$ ) on the potential outcomes. For example, in a trial where patients experience a maximum of two episodes, we might assume the following model

(2) $$Y_{ij}=\alpha +\beta Z_{ij}+\gamma Z_{i,j-1}+\delta Z_{ij}Z_{i,j-1}+\beta_{ep}X_{ep_{ij}}+\varepsilon_{ij}$$

where $Z_{i,j-1}$ is the treatment allocation in the previous episode (and is set to 0 for j=1 ), and $X_{ep_{ij}}$ is an indicator for episode 2 (i.e. $X_{ep_{ij}}=1$ for episode 2, and 0 otherwise). This model allows the effect of the intervention in episode 1 to carry forward into episode 2 (the term γ ), and for the intervention to get more (or less) effective the second time, it is used (the term δ ).

After obtaining estimates for β , γ , and δ , we can use these to estimate the $\beta_{ij}^{PB}$ 's; for example, following on from the definition of the per-episode policy-benefit estimand and model (2) above, the ${\hat{\beta }}_{ij}^{PB}$ for all first episodes is $\hat{\beta }$ , and for all second episodes is $\hat{\beta }+\hat{\gamma }+\hat{\delta }$ .

We can then use these estimates to get an overall estimate of $\beta_{E}^{PB}$ as follows

$${\hat{\beta }}_{E}^{PB}=\frac{N_{1}}{M_{T}}(\hat{\beta })+\frac{N_{2}}{M_{T}}(\hat{\gamma }+\hat{\beta }+\hat{\delta })$$

Although the term $\beta_{ep}$ is not directly used to estimate the treatment effect, it is necessary to include $X_{ep_{ij}}$ in model (2), as estimates may be biased otherwise. This is because $X_{ep_{ij}}$ is associated with $Z_{i,j-1}$ , and so may act as a confounder if omitted from the model, resulting in biased estimates of γ . For trials with j>2 , separate indicator variables for each episode should be included in the model.

This estimator will be unbiased if (a) we correctly specify the causal model in (2); and if (b) we are able to obtain unbiased estimates of the causal parameters (e.g. β , γ , and δ in model (2)). In some instances, this estimator will be unbiased even if we misspecify the causal model, for instance by including an additional unneeded parameter (e.g. including $Z_{i,j-1}$ in the model when potential outcomes in the current episode are not affected by treatment history). However, it will generally be biased if we omit a true causal parameter from the model (e.g. if we omit $Z_{i,j-1}$ from the model if potential outcomes are affected by treatment history).

We note that even if the causal model in (2) is correctly specified, we may still obtain biased estimates of the causal parameters; for example, under differential non-enrolment, the parameter estimate of γ will be biased, and hence the overall estimator will also be biased.^15^

### 4.4 Per-patient policy-benefit estimator

For the per-patient policy-benefit estimator we need to obtain estimates from model (2) using weighted least-squares, where each patient is weighted by the inverse of their number of episodes, $W_{i}=\frac{1}{M_{i}}$ . After obtaining estimates and calculating the ${\hat{\beta }}_{ij}^{PB}$ 's, the overall treatment effect is calculated as

$${\hat{\beta }}_{P}^{PB}=\frac{1}{N_{T}}\underset{ij}{\sum }\;W_{i}{\hat{\beta }}_{ij}^{PB}$$

Using analysis model (2) above, this equates to

$${\hat{\beta }}_{P}^{PB}=\frac{M_{T(1)}}{N_{T}}(\hat{\beta })+\frac{M_{T(2)}}{N_{T}}\left(\frac{1}{2}\hat{\beta }+\left(\frac{1}{2}\right)(\hat{\beta }+\hat{\gamma }+\hat{\delta })\right)$$

where $M_{T(j)}$ represents the total number of patients for whom $M_{i}=j$ (e.g. $M_{T(3)}=30$ denotes that 30 patients were enrolled for three episodes). In this equation, $\frac{M_{T(1)}}{N_{T}}(\hat{\beta })$ is the component for patients, where $M_{i}=1$ , and $\frac{M_{T(2)}}{N_{T}}\left(\frac{1}{2}\hat{\beta }+\left(\frac{1}{2}\right)(\hat{\beta }+\hat{\gamma }+\hat{\delta })\right)$ is the component for patients, where $M_{i}=2$ (with $\frac{1}{2}\hat{\beta }$ being the first episode component, and $\left(\frac{1}{2}\right)(\hat{\beta }+\hat{\gamma }+\hat{\delta })$ being the second episode component).

This estimator will be biased in the same settings as the per-episode policy-benefit estimator^15^ (incorrectly specified causal model, biased parameter estimates due to differential non-enrolment), and in the same settings as the per-patient added-benefit estimator (differential non-enrolment based on the outcome from the previous episode).

## 5 Example: Trial in acute sickle cell pain crises

An example of a setting where re-randomisation has been used is trials in acute pain crises in patients with sickle cell disease.^2^ Pain crises can be recurring, and so some patients may experience more than one pain crisis in a given period of time.^5^

Patients who experience sickle cell pain crises typically require hospitalisation and treatment to manage symptoms. Treatment generally consists of morphine, which can have unwanted side effects. Therefore, alternative treatment options would be useful; for example, one trial assessed the use of high-dose ibuprofen compared to a placebo to reduce the amount of morphine required to manage pain symptoms.^31^ We note that the interventions under study in this example (ibuprofen, placebo) will not affect the occurrence of future episodes (e.g. ibuprofen would not prevent future pain crises from occurring).

There are two important considerations when choosing the most estimand. First, the distribution of pain crises across patients is often skewed, where most patients may experience only one or two episodes during the course of the trial, but a small number of patients will experience a much larger number of episodes.^5^ Therefore, differences between the per-episode and per-patient estimands may be large if the treatment effect differs in patients predisposed to experience a large number of pain crises. Both estimands address clinically important questions. The per-episode has the interpretation ‘the average effect across each time the intervention is used’. It gives more weight to patients who experience frequent pain crises, and it could be argued that it is more important that the intervention work well in these patients compared to those who experience infrequent crises (as they will undergo the intervention more frequently). However, it may also be useful to know how well the intervention works for the average patient; for instance, if the intervention works well for the 80% of patients in the trial who experience only one or two pain crises, this would still be clinically important to know, even if it worked less well in the other 20% of patients who experienced more frequent pain crises. Therefore, both estimands could be used, one as the primary and the other as a secondary estimand. Given that per-episode effects can be estimated under less restrictive assumptions (i.e. it does not require the assumption of no differential non-enrolment, unlike the per-patient estimator), we suggest this could be considered for the primary estimand, though this choice largely depends on the overall goals of the trial.

Second, the interventions used to treat symptoms from acute sickle cell pain crises (e.g. ibuprofen^5^) are unlikely to influence either the outcome or treatment effect in subsequent episodes. Therefore, the added-benefit and policy-benefit estimands should be similar. Given the additional complexity of estimating policy-benefit effects, we suggest an added-benefit approach be used here. Coupled with a per-episode approach, this estimand provides an interpretation of ‘the additional benefit of the intervention averaged across all pain crises for which it would be used’, and can be easily estimated using the difference between all interventions episodes versus all control episodes.

## 6 Discussion

We have proposed a set of estimands that can be used in multi-episode settings, as well as a set of independence estimators that can be used in re-randomisation trials. Our main result is to show that the analytical approach most commonly used in re-randomisation trials (comparing all intervention vs. all control episodes directly) corresponds to a per-episode added-benefit estimand. This implies these trials have been estimating the average effect of the intervention across episodes, over and above any benefit conferred from the intervention in previous episodes. We have found the per-episode added-benefit estimator to be generally unbiased, which suggests results from trials using this approach are valid.

However, other estimands we have proposed here may also be useful in future trials. For instance, in trials for which some patients experience a large number of episodes, per-patient estimands may give a better idea of how well the intervention works for the average patient in such settings. Similarly, in trials for which the treatments are expected to influence outcomes or treatment effects in subsequent episodes, policy-benefit estimands may provide a better picture of the overall benefit to adopting such interventions into routine practice.

Our derivations show that while the per-episode added-benefit estimator is generally unbiased in re-randomisation trials, other estimands (per-patient added-benefit and policy-benefit estimands) require untestable assumptions for unbiasedness. Sometimes we can determine that the assumptions are plausible based on subject matter knowledge (e.g. the assumptions underpinning the policy-benefit estimator in the sickle cell example given earlier), but in other settings, the required assumptions might be quite strong (e.g. it may be unlikely we are able to determine an appropriate causal model for the policy-benefit estimator under complex carryover mechanisms, or when carryover or treatment effects interact with disease progression). In these settings, it may be desirable to specify policy-benefit or per-patient as secondary estimands, with the per-episode added-benefit used as the primary (as it can be reliably estimated without strong assumptions). Alternatively, if policy-benefit is the main question of interest and it is anticipated that a plausible causal model cannot be identified, then it may be useful to consider alternative trial designs which can more easily estimate this type of estimand, such as a cluster design where participants are assigned to receive the same treatment for each episode; however, only limited research has been undertaken on this type of trial design to date, and further research is warranted before it is used regularly.

One drawback of the added-benefit estimand is that it averages over different (randomised) treatment histories, which will not reflect the treatment histories seen in clinical practice. If the treatment effect is not modified by treatment allocation in previous episodes (as would be expected in the sickle cell example given earlier), then the added-benefit estimand will generalise to clinical practice, as the treatment histories will not affect the value of the estimand. Otherwise, the added-benefit estimand will be less generalisable, though depending on the specific study objectives, it may still be useful (as it is the only way of addressing the question ‘how much additional benefit is conferred by the intervention in *this* episode, over and above any previous episodes’ which can be estimated without strong assumptions).

As with any treatment effect which is an average over different patients or data points, the estimands defined here may not be representative of the treatment effect for a particular episode. For instance, the treatment effect may decrease in later episodes compared to earlier ones (for instance, in the case of progressive disease, where patients’ health status gradually worsens for each new episode they experience, and there is a treatment-by-disease status interaction, or if the treatment itself loses efficacy the more often it is given). It may be of interest to explore supplementary estimands here, particularly to help identify whether treatment should be given for each new episode, or whether there is a certain point at which it becomes no longer useful. This could be done using episode-specific estimands (i.e. the treatment effect the first time a treatment is given vs. the second time, etc.), though we note this may not be sufficient to answer the question on its own. For instance, consider the example of progressive disease given above; the episode-specific effects will show the treatment becoming less effective in later episodes, though it won’t be clear whether this is due to the treatment itself becoming less effective, or because of an interaction with disease progression. Therefore, in this type of setting it may also be worth exploring subgroup analyses (e.g. the episode-specific effect in patients whose disease has progressed vs. those where it has not), which may provide a more complete picture of which patients or episodes should receive treatment.

The re-randomisation design is a new type of trial design, and as such, there has been little methodological research to date. Future extensions to the work in this article would be useful, for instance, to evaluate alternatives to independence estimators (such as mixed-effects models), or alternative designs (such as a cluster design where patients are allocated to receive the same treatment for all episodes). Sample size requirements will likely differ for each of the estimands described (e.g. sample size requirements for the per-patient estimands will likely be based on recruiting a specified number of patients, whereas the per-episode estimands will likely require a specified number of episodes, irrespective of patients^5^), and so further research in this area is warranted. Further work to implement new packages in routine statistical software (such as Stata or R) to automate estimation of the policy-benefit estimands (which can be challenging to implement manually, due to the need to specify a different causal model depending on the number of episodes, and then to average over different parameters in the causal model) would be useful. More generally, further work on the estimation of policy-benefit estimands would be useful, particularly in the setting where some patients experience a large number of episodes, where including an indicator variable for each episode may lead to computational issues. Finally, in this paper, we considered the setting where treatment allocation does not affect the occurrence of subsequent episodes. In some clinical settings, this may not be the case, and so it would be useful to extend the estimands here to the setting where treatment allocation may prevent subsequent episodes from occurring.

## 7 Conclusions

Consideration of the proposed estimands can help guide the choice of an appropriate analytical method. The re-randomisation design in conjunction with an independence estimator can be a useful approach in multi-episode settings.

## Supplemental Material

sj-docx-1-smm-10.1177_09622802221094140 - Supplemental material for Re-randomisation trials in multi-episode settings: Estimands and independence estimators Supplemental material, sj-docx-1-smm-10.1177_09622802221094140 for Re-randomisation trials in multi-episode settings: Estimands and independence estimators by Brennan C Kahan, Ian R White, Richard Hooper and Sandra Eldridge in Statistical Methods in Medical Research
