# Supplementary material for: Re-randomisation trials in multi-episode settings: Estimands and independence estimators
Source: Stat Methods Med Res. 2022 Apr 14;31(7):1342–54. doi: 10.1177/09622802221094140 (PMC9251752; doi:10.1177/09622802221094140)
Supplement: sj-docx-1-smm-10.1177_09622802221094140 - Supplemental material for Re-randomisation trials in multi-episode settings: Estimands and independence estimators [file sj-docx-1-smm-10.1177_09622802221094140.docx]

**Suppementary material for:** ***Re-randomisation trials in multi-episode settings: estimands and independence estimators***

Brennan C Kahan, Ian R White, Richard Hooper, Sandra Eldridge

**Section 1 - Overview**

This this supplementary material we (a) show how the estimands differ under various data generating mechanisms (section 3); and (b) evaluate bias for the per-episode added-benefit and per-patient added-benefit estimators under a range of scenarios (section 4). In section 2 we provide some additional notation needed for these derivations.

For simplicity, we show our key results below: Table 1 shows the estimand differences under different data generating mechanisms, and Tables 2 and 3 show the expected value of the per-episode and per-patient added-benefit estimators against the true estimand values (these results are derived in sections 3 and 4). We discuss these results in sections 3 and 4.

**Table 1 - Treatment estimands under different treatment effect mechanisms in a fictitious trial with a maximum of two episodes.** $\boldsymbol{p}$ **denotes the proportion of patients in the trial who enrolled for two episodes**

| **Scenario** | **Data generating model** | **Per-episode added-benefit (**$\boldsymbol{\beta}_{\boldsymbol{E}}^{\boldsymbol{AB}}$**)** | **Per-episode policy-benefit (**$\boldsymbol{\beta}_{\boldsymbol{E}}^{\boldsymbol{PB}}$**)** | **Per-patient added-benefit (**$\boldsymbol{\beta}_{\boldsymbol{P}}^{\boldsymbol{AB}}$**)** | **Per-patient policy-benefit (**$\boldsymbol{\beta}_{\boldsymbol{P}}^{\boldsymbol{PB}}$**)** |
| --- | --- | --- | --- | --- | --- |
| Constant treatment effect | $Y_{ij}=\alpha+\beta Z_{ij}+\mu_{i}+\varepsilon_{ij}$ | $\beta$ | $\beta$ | $\beta$ | $\beta$ |
| Treatment effect varies across episodes | $Y_{ij}=\left\{ \begin{aligned} \alpha+\beta_{1}Z_{ij}+\mu_{i}+\varepsilon_{ij}\mathrm{for}j=1 \\ \alpha+\beta_{2}Z_{ij}+\mu_{i}+\varepsilon_{ij}\text{ for }j=2 \end{aligned} \right.$ | $\frac{\beta_{1}+p\beta_{2}}{1+p}$ | $\frac{\beta_{1}+p\beta_{2}}{1+p}$ | $\left( 1-\frac{p}{2} \right)\beta_{1}+\frac{p}{2}\beta_{2}$ | $\left( 1-\frac{p}{2} \right)\beta_{1}+\frac{p}{2}\beta_{2}$ |
| Treatment effect varies across patients with different values of $M_{i}$ | $Y_{ij}=\left\{ \begin{aligned} \alpha+\beta_{1}Z_{ij}+\mu_{i}+\varepsilon_{ij}\mathrm{if}M_{i}=1 \\ \alpha+\beta_{2}Z_{ij}+\mu_{i}+\varepsilon_{ij}\text{ if }M_{i}=2 \end{aligned} \right.$ | $\frac{\left( 1-p \right)\beta_{1}+2p\beta_{2}}{\left( 1+p \right)}$ | $\frac{\left( 1-p \right)\beta_{1}+2p\beta_{2}}{\left( 1+p \right)}$ | $\left( 1-p \right)\beta_{1}+p\beta_{2}$ | $\left( 1-p \right)\beta_{1}+p\beta_{2}$ |
| Treatment effect carries forward | $Y_{ij}=\alpha+\beta Z_{ij}+\gamma Z_{i,j-1}+\mu_{i}+\varepsilon_{ij}$ | $\beta$ | $\beta+\frac{p\gamma}{\left( 1+p \right)}$ | $\beta$ | $\beta+\frac{p\gamma}{2}$ |
| Treatment becomes less effective on re-use | $Y_{ij}=\left\{ \begin{aligned} \alpha+\beta Z_{ij}+\mu_{i}+\varepsilon_{ij}\text{ for }Z_{i,j-1}=0 \\ \alpha+\left( \beta+\delta\right)Z_{ij}+\mu_{i}+\varepsilon_{ij}\text{ for }Z_{i,j-1}=1 \end{aligned} \right.$ | $\beta+\frac{p\delta}{2\left( 1+p \right)}$ | $\beta+\frac{p\delta}{\left( 1+p \right)}$ | $\beta+\frac{p\delta}{4}$ | $\beta+\frac{p\delta}{2}$ |

**Table 2 - Summary of mathematical derivations for the per-episode added-benefit independence estimator**

| **Scenario** | $\boldsymbol{E}\left( {\hat{\boldsymbol{\beta}}}_{\boldsymbol{E}}^{\boldsymbol{AB}} \right)$ | **True value of estimand** |
| --- | --- | --- |
| S1 – Constant treatment effect | $\beta$ | $\beta$ |
| S2 – Treatment effect varies across episode | $\frac{\beta_{1}+p\beta_{2}}{1+p}$ | $\frac{\beta_{1}+p\beta_{2}}{1+p}$ |
| S3 – Treatment effect varies across value of $M_{i}$ | $\frac{\left( 1-p \right)\beta_{1}+2p\beta_{2}}{\left( 1+p \right)}$ | $\frac{\left( 1-p \right)\beta_{1}+2p\beta_{2}}{\left( 1+p \right)}$ |
| S4 – Treatment effect carries forward into second episode | $\beta$ | $\beta$ |
| S5 – Treatment becomes less effective on re-use | $\beta+\frac{p\delta}{2\left( 1+p \right)}$ | $\beta+\frac{p\delta}{2\left( 1+p \right)}$ |
| S6 – Constant treatment effect, differential non-enrolment based on outcome in previous episode | $\beta_{trt}$ | $\beta_{trt}$ |
| S7 – Constant treatment effect, differential non-enrolment based on expected outcome in current episode | $\beta_{trt}$ | $\beta_{trt}$ |

**Table 3 - Summary of mathematical derivations for the per-patient added-benefit independence estimator**

| **Scenario** | $\boldsymbol{E}\left( {\hat{\boldsymbol{\beta}}}_{\boldsymbol{P}}^{\boldsymbol{AB}} \right)$ | **True value of estimand** |
| --- | --- | --- |
| S1 – Constant treatment effect | $\beta$ | $\beta$ |
| S2 – Treatment effect varies across episode | $\left( 1-\frac{p}{2} \right)\beta_{1}+\frac{p}{2}\beta_{2}$ | $\left( 1-\frac{p}{2} \right)\beta_{1}+\frac{p}{2}\beta_{2}$ |
| S3 – Treatment effect varies across value of $M_{i}$ | $\left( 1-p \right)\beta_{1}+p\beta_{2}$ | $\left( 1-p \right)\beta_{1}+p\beta_{2}$ |
| S4 – Treatment effect carries forward into second episode | $\beta$ | $\beta$ |
| S5 – Treatment becomes less effective on re-use | $\beta+\frac{p\delta}{4}$ | $\beta+\frac{p\delta}{4}$ |
| S6 – Constant treatment effect, differential non-enrolment based on outcome in previous episode | $\beta_{trt}+\beta_{X_{PL}}\frac{\left( p_{01}-p_{00} \right)}{4}$ | $\beta_{trt}$ |
| S7 – Constant treatment effect, differential non-enrolment based on expected outcome in current episode | $\beta_{trt}$ | $\beta_{trt}$ |

**Section 2 - Notation**

A summary of key notation is provided in Table 4.

Let $R_{ij}$ denote whether patient $i$ is enrolled in the trial at episode $j$ (where $R_{ij}=1$ means the patient was enrolled, and $R_{ij}=0$ means they were not enrolled; note that $R_{ij}=0$ if $M_{i}<j$), and let $R_{ij}^{\left( \tilde{Z}=\tilde{z} \right)}$ denote the patient’s potential enrolment status at episode $j$ under treatment history $\tilde{Z}_{ij}=\tilde{z}_{ij}$. Then, $P\left( \tilde{Z}_{ij}=\tilde{z}_{ij} \right)=0$ if $R_{ij}^{\left( \tilde{Z}=\tilde{z} \right)}=0$(i.e. if patient $i$ would not be enrolled at episode $j$ under treatment history $\tilde{Z}_{ij}=\tilde{z}_{ij}$, then the probability of this treatment history being observed for this patient at episode $j$ is 0). Under a 1:1 allocation ratio, all treatment histories $\tilde{Z}_{ij}=\tilde{z}_{ij}$ for which $R_{ij}^{\left( \tilde{Z}=\tilde{z} \right)}=1$ are equally like to be observed. Note that $\sum_{\tilde{Z}_{ij}} P \left( \tilde{Z}_{ij}=\tilde{z}_{ij} \right)=1$.

**Table 4 - Summary of notation**

| **Notation** | **Definition** |
| --- | --- |
| *Notation related to number of episodes/patients* | |
| $M_{i}$ | The number of episodes for which patient $i$ is enrolled in the trial |
| $M_{T\left( j \right)}$ | The total number of patients for whom $M_{i}=j$ |
| $M_{T}$ | The total number of episodes enrolled in the trial |
| $N_{j}$ | The number of patients who are enrolled in the trial for at least $j$ episodes |
| $N_{T}$ | The total number of patients enrolled in the trial |
| *Variables* | |
| $Y_{ij}$ | Outcome for patient $i$ during episode $j$ |
| $Z_{ij}$ | Treatment allocation for patient $i$ during episode $j$ (where 0=control, 1=intervention) |
| $Z_{i,j-1}$ | Treatment allocation in the patient's previous episode (defined as 0 for $j=1$) |
| $\tilde{Z}_{ij}$ | A vector of previous treatment allocations for patient $i$ (for example, $\tilde{Z}_{13}$ would be the vector $\left( Z_{11},Z_{12} \right)$); this is referred to as the ‘treatment history’ |
| *Notation related to sampling of episodes/patients (used for estimands)* | |
| $I^{E}$ | A random variable, where $I^{E}=i$ with probability $\frac{M_{i}}{M_{T}}$ |
| $J^{E}$ | A random variable, which, conditional on $I^{E}$ has a uniform distribution on $\left( 1,\ldots,M_{I^{E}} \right)$ |
| $\left( IJ \right)^{E}$ | Represents $I^{E}J^{E}$, which represents a randomly selected episode from within the trial, where each episode has an equal probability of being selected ($\frac{1}{M_{T}}$) |
| $Y_{\left( IJ \right)^{E}}$ | The outcome for a randomly selected episode in the trial |
| $Z_{\left( IJ \right)^{E}}$ | The treatment allocation for a randomly selected episode in the trial |
| $\tilde{Z}_{\left( IJ \right)^{E}}$ | The treatment history for a randomly selected episode in the trial |
| $I^{P}$ | A random variable which has a uniform distribution on $\left( 1,\ldots,N_{T} \right)$ |
| $J^{P}$ | A random variable, which, conditional on $I^{P}$ has a uniform distribution on $\left( 1,\ldots,M_{I^{P}} \right)$ |
| $\left( IJ \right)^{P}$ | Represents $I^{P}J^{P}$, which represents a randomly selected episode from a randomly selected patient from within the trial, where each patient has a probability $\frac{1}{N_{T}}$ of being selected, and each episode from the chosen patient has a probability $\frac{1}{M_{I^{P}}}$ of being selected |
| $Y_{\left( IJ \right)^{P}}$ | The outcome for a randomly selected episode from a randomly selected patient |
| $Z_{\left( IJ \right)^{P}}$ | The treatment allocation for a randomly selected episode from a randomly selected patient |
| $\tilde{Z}_{\left( IJ \right)^{P}}$ | The treatment history for a randomly selected episode from a randomly selected patient |

**Section 3 - Differences between estimands in a fictitious example**

We illustrate the similarities and differences between the estimands using a simple fictitious example. Consider a trial where patients experience a maximum of two episodes, with a 1:1 allocation ratio. Further, assume there is no non-enrolment (i.e. patients who experience two episodes enrol in the trial for both episodes, that is, $R_{i2}=1$ for all patients with $M_{i}=2$). Let $p$ denote the proportion of patients in the trial who enrolled for two episodes (i.e. $p=\frac{M_{T\left( 2 \right)}}{N_{T}}$). We evaluate the estimands under a variety of different generating mechanism using the formulas for estimands described in the main article.

We evaluate the estimands under five different data generating models (which we refer to as ‘treatment effect scenarios’), shown in Table 1. These are: (a) constant treatment effect (the treatment effect is the same across all patients and episodes); (b) the treatment effect varies across episodes (the treatment effect is different in episode 1 vs. episode 2); (c) the treatment effect varies across patients depending on whether $M_{i}=1$ vs. $M_{i}=2$ (the treatment effect is different in patients who require treatment less often vs. more often); (d) the treatment effect carries forward (patients who received the intervention in their 1st episode have different outcomes in their 2nd episode compared to those who received control in their 1st episode); and (e) the treatment becomes less effective on re-use (patients who received the intervention in their 1st episode have different treatment effects in their 2nd episode compared to those who received control in their 1st episode).

In each data generating model, $\mu_{i}$ represents a patient-specific random-intercept, and $\varepsilon_{ij}$ represents an episode-level random error term.

Values for the different estimands under different treatment effect scenarios are shown in Table 1.

All four estimands coincide under a constant treatment effect mechanism; this is because the cluster size is not informative (i.e. the potential treatment effects $\bar{\beta}_{ij}^{AB}$ and $\beta_{ij}^{PB}$ do not differ according to $M_{i}$), and the potential outcomes and potential treatment effects ($Y_{ij}^{\left( Z=0,\tilde{Z}=\tilde{z} \right)}$ and $Y_{ij}^{\left( Z=1,\tilde{Z}=\tilde{z} \right)}-Y_{ij}^{\left( Z=0,\tilde{Z}=\tilde{z} \right)}$) are not affected by $\tilde{Z}_{ij}$.

When the treatment effect varies across episodes or across patients with different values of $M_{i}$, the per-episode and per-patient estimands differ (because the cluster size is informative), though the added-benefit and policy-benefit estimands do not (because treatment history does not affect either the potential outcomes or potential treatment effects).

When the treatment effect carries forward, the per-episode added-benefit and per-patient added-benefit estimands coincide, because the $\bar{\beta}_{ij}^{AB}$ treatment effect does not differ according to $M_{i}$. However, the per-episode and per-patient policy-benefit estimands differ; this is because the $\beta_{ij}^{PB}$ treatment effects do differ according to $M_{i}$.

Finally, when the intervention becomes less effective on re-use, all four estimands differ. This is because both sets of potential treatment effects ($\bar{\beta}_{ij}^{AB}$ and $\beta_{ij}^{PB}$) differ according to $M_{i}$, and the potential treatment effects ($Y_{ij}^{\left( Z=1,\tilde{Z}=\tilde{z} \right)}-Y_{ij}^{\left( Z=0,\tilde{Z}=\tilde{z} \right)}$) are affected by $\tilde{Z}_{ij}$.

**Section 4 - Mathematical derivation of bias for per-episode and per-patient added-benefit estimators**

In this section we evaluate the bias of independence estimators for the per-episode and per-patient added-benefit estimands. Results are shown in Tables 2 and 3. For simplicity, we restrict ourselves to a trial with a 1:1 allocation ratio, where patients experience a maximum of two episodes. As before, we assume that treatment allocation does not affect the occurrence of subsequent episodes.

We first discuss the number of patients belonging to each different treatment sequence that could occur in a re-randomisation trial in this setting (as this will be used in the derivation of the expected value of the independence estimators). Then, we derive the expected value of the independence estimators. We then compare the expected values against the true estimand values (given in Table 1) under a range of different data generating and non-enrolment mechanisms.

**Number of patients at each episode for different sequences**

Assuming there is no non-enrolment (i.e. patients enrol in the trial for each episode they experience), the number of episodes in each treatment sequence that occur asymptotically under a 1:1 allocation ratio are shown in Table 5 (where $p$ represents the proportion of patients in the trial who experience two episodes). The assumption of no non-enrolment will be relaxed in later sections.

In this setting, there are six possible treatment sequences (shown in Table 5). Let $Z$ denote the treatment sequence; i.e. $Z$ is one of $(0)$, $(1)$, $(0,0)$, $(0,1)$, $(1,0)$, or $(1,1)$. There are $N_{T}\left( 1-p \right)$ patients enrolled for a single episode; therefore, there are $\frac{N_{T}}{2}\left( 1-p \right)$ patients in treatment sequences $Z=\left( 0 \right)$ and $Z=(1)$ respectively. There are $N_{T}p$ patients enrolled for two episodes; therefore, there are $\frac{N_{T}p}{4}$ patients in treatment sequences $Z=(0,0)$, $Z=(0,1)$, $Z=(1,0)$, and $Z=(1,1)$.

**Table 5 - Asymptotic number of patients in each treatment sequence at each episode in a re-randomisation trial with 1:1 allocation ratio and no non-enrolment**

|  | **Treatment allocation** | | **Number of patients** | |
| --- | --- | --- | --- | --- |
| Sequence | Episode 1 | Episode 2 | Episode 1 | Episode 2 |
| $Z=(1)$ | 1 | $-$ | $\frac{N_{T}}{2}\left( 1-p \right)$ | $-$ |
| $Z=(1,0)$ | 1 | 0 | $\frac{N_{T}p}{4}$ | $\frac{N_{T}p}{4}$ |
| $Z=(1,1)$ | 1 | 1 | $\frac{N_{T}p}{4}$ | $\frac{N_{T}p}{4}$ |
| $Z=(0)$ | 0 | $-$ | $\frac{N_{T}}{2}\left( 1-p \right)$ | $-$ |
| $Z=(0,1)$ | 0 | 1 | $\frac{N_{T}p}{4}$ | $\frac{N_{T}p}{4}$ |
| $Z=(0,0)$ | 0 | 0 | $\frac{N_{T}p}{4}$ | $\frac{N_{T}p}{4}$ |

**Expected values of independence estimators**

***Between- and within-patient estimation components***

The treatment sequences in Table 5 can be split into three different estimation components; two are between-patient estimation components and one is a within-patient estimation component (1). These estimation components are used as the basis for the mathematical derivations in this section.

Let $\hat{\beta}_{B_{1}}$ represent the first between-patient estimation component; it is based on treatment sequences $Z=(0)$ and $Z=(1)$. It uses between-patient information for patients who are enrolled for one episode. It is calculated as:

$$\hat{\beta}_{B_{1}}=\frac{\sum_{i\in Z=\left( 1 \right)} Y_{i1}}{\frac{N_{T}}{2}\left( 1-p \right)}-\frac{\sum_{i\in Z=\left( 0 \right)} Y_{i1}}{\frac{N_{T}}{2}\left( 1-p \right)}=\frac{1}{\frac{N_{T}}{2}\left( 1-p \right)}\left( \sum_{i\in Z=\left( 1 \right)} Y_{i1}-\sum_{i\in Z=\left( 0 \right)} Y_{i1} \right)$$

i.e. it is the mean of all episodes in treatment sequence $Z=(1)$ vs. the mean of all episodes in treatment sequence $Z=(0)$ (where the denominators are from Table 5).

Let $\hat{\beta}_{B_{2}}$ represent the second between-patient estimation component; it is based on treatment sequences $Z=\left( 0, 0 \right)$ and $Z=\left( 1, 1 \right)$. It uses between-patient information for patients who are enrolled for two episodes and allocated to the same treatment for each episode. It is calculated as:

$$\hat{\beta}_{B_{2}}=\frac{\sum_{i\in Z=\left( 1,1 \right)} \left( Y_{i1}+Y_{i2} \right)}{\frac{N_{T}p}{4}+\frac{N_{T}p}{4}}-\frac{\sum_{i\in Z=\left( 0,0 \right)} \left( Y_{i1}+Y_{i2} \right)}{\frac{N_{T}p}{4}+\frac{N_{T}p}{4}}=\frac{1}{\frac{N_{T}p}{2}}\left( \sum_{i\in Z=\left( 1,1 \right)} \left( Y_{i1}+Y_{i2} \right)-\sum_{i\in Z=\left( 0,0 \right)} \left( Y_{i1}+Y_{i2} \right) \right)$$

i.e. it is the mean of all episodes in treatment sequence $Z=\left( 1, 1 \right)$ vs. the mean of all episodes in treatment sequences $Z=\left( 0, 0 \right)$.

Finally, let $\hat{\beta}_{W}$ represents the within-patient estimation component; it is based on treatment sequences $Z=\left( 0, 1 \right)$ and $Z=\left( 1, 0 \right)$. It uses within-patient information for patients who are enrolled for two episodes and allocated to different treatments for each episode. It is calculated as:

$$\hat{\beta}_{W} =\frac{\sum_{i\in Z=\left( 1,0 \right)} \left( Y_{i1}-Y_{i2} \right)}{\frac{N_{T}p}{4}+\frac{N_{T}p}{4}}-\frac{\sum_{i\in Z=\left( 0,1 \right)} \left( Y_{i1}-Y_{i2} \right)}{\frac{N_{T}p}{4}+\frac{N_{T}p}{4}}=\frac{1}{\frac{N_{T}p}{2}}\left( \sum_{i\in Z=\left( 1,0 \right)} \left( Y_{i1}-Y_{i2} \right)-\sum_{i\in Z=\left( 0,1 \right)} \left( Y_{i1}-Y_{i2} \right) \right)$$

i.e. it is the mean of intervention episodes for patients on treatment sequences $Z=\left( 0, 1 \right)$ and $Z=\left( 1, 0 \right)$ vs. the mean of control episodes for patients on treatment sequences $Z=\left( 0, 1 \right)$ and $Z=\left( 1, 0 \right)$.

***Per-episode added-benefit estimator***

This estimator can be written in terms of the components $\hat{\beta}_{B_{1}}$, $\hat{\beta}_{B_{2}}$, and $\hat{\beta}_{W}$, as:

$$\hat{\beta}_{E}^{AB}=\frac{\sum_{ij} Y_{ij}Z_{ij}}{\sum_{ij} Z_{ij}} -\frac{\sum_{ij} Y_{ij}\left( 1-Z_{ij} \right)}{\sum_{ij} \left( 1-Z_{ij} \right)}=\frac{\frac{N_{T}}{2}\left( 1-p \right)\hat{\beta}_{B_{1}}+\frac{N_{T}p}{2}\hat{\beta}_{B_{2}}+\frac{N_{T}p}{2}\hat{\beta}_{W}}{\frac{N_{T}}{2}\left( 1-p \right)+\frac{N_{T}p}{2}+\frac{N_{T}p}{2}}=\frac{\left( 1-p \right)\hat{\beta}_{B_{1}}+p\hat{\beta}_{B_{2}}+p\hat{\beta}_{W}}{1+p} (1)$$

Taking the expectation leads to:

$$E\left( \hat{\beta}_{E}^{AB} \right)=\frac{\left( 1-p \right)E\left( \hat{\beta}_{B_{1}} \right)pE+\left( \hat{\beta}_{B_{2}} \right)pE+\left( \hat{\beta}_{W} \right)}{1+p}$$

***Per-patient added-benefit estimator***

This estimator can also be written in terms of the components $\hat{\beta}_{B_{1}}$, $\hat{\beta}_{B_{2}}$, and $\hat{\beta}_{W}$, by using equation (1) above and weighting each component by $\frac{1}{M_{i}}$:

$$\hat{\beta}_{P}^{AB}=\frac{\sum_{ij} W_{i}Y_{ij}Z_{ij}}{\sum_{ij} W_{i}Z_{ij}}-\frac{\sum_{ij} W_{i}Y_{ij}\left( 1-Z_{ij} \right)}{\sum_{ij} W_{i}\left( 1-Z_{ij} \right)}=\frac{\left( \frac{1}{1} \right)\left( \frac{N_{T}}{2} \right)\left( 1-p \right)\hat{\beta}_{B_{1}}+\left( \frac{1}{2} \right)\left( \frac{N_{T}p}{2} \right)\hat{\beta}_{B_{2}}+\left( \frac{1}{2} \right)\left( \frac{N_{T}p}{2} \right)\hat{\beta}_{W}}{\left( \frac{1}{1} \right)\left( \frac{N_{T}}{2} \right)\left( 1-p \right)+\left( \frac{1}{2} \right)\left( \frac{N_{T}p}{2} \right)+\left( \frac{1}{2} \right)\left( \frac{N_{T}p}{2} \right)}=\left( 1-p \right)\hat{\beta}_{B_{1}}+\frac{p}{2}\hat{\beta}_{B_{2}}+\frac{p}{2}\hat{\beta}_{W}$$

Taking the expectation leads to:

$$E\left( \hat{\beta}_{P}^{AB} \right)=\left( 1-p \right)E\left( \hat{\beta}_{B_{1}} \right)+\frac{p}{2}E\left( \hat{\beta}_{B_{2}} \right)+\frac{p}{2}E\left( \hat{\beta}_{W} \right)$$

**Expected value of estimators under different data generating mechanisms and non-enrolment scenarios**

We now evaluate the expected values of the per-episode and per-patient added-benefit estimators under a range of data generating and non-enrolment scenarios. We evaluate seven scenarios in total:

- Constant treatment effect
- Treatment effect varies across episode
- Treatment effect varies across value of $M_{i}$
- Treatment effect carries forward into second episode
- Treatment becomes less effective on re-use
- Constant treatment effect, differential non-enrolment based on outcome in previous episode
- Constant treatment effect, differential non-enrolment based on expected outcome in current episode

Exact data generating mechanisms are shown in later sections. The first five scenarios do not involve any non-enrolment (i.e. patients who experience two episodes will enrol in the trial for both episodes). The last two scenarios use a constant treatment effect mechanism, but some patients who experience two episodes do not re-enrol for their second episode. In these two scenarios we examine the impact of differential non-enrolment. In this paper we have defined differential non-enrolment to mean that different types of patients from the episode 1 intervention and control groups will re-enrol for episode 2. For example, in the episode 1 intervention group healthier patients are more likely to re-enrol than sicker patients, but in the episode 1 control group sicker patients are more likely to re-enrol. In scenario 6, non-enrolment is differential between treatment arms depending on their outcome in the first episode; patients who received the intervention in episode 1 and had a good outcome have the same probability of re-enrolling as patients who received control in episode 1 and had a bad outcome, and vice versa. In scenario 7, non-enrolment is differential between treatment arms depending on their expected outcome in the second episode (i.e. their baseline prognosis at episode 2); patients who received the intervention in episode 1 and have a good baseline prognosis at episode 2 have the same probability of re-enrolling as patients who received control in episode 1 and had a bad prognosis, and vice versa. Further details on each of these scenarios is given in the sections below.

For each scenario, we will assume that $\alpha=0$ (i.e. the intercept value in data generating models is 0). Expected values of the components $\hat{\beta}_{B_{1}}$, $\hat{\beta}_{B_{2}}$, and $\hat{\beta}_{W}$ under the different scenarios are shown in Table 6 (these are derived in the subsections below for each scenario).

**Table 6 - Expected values of between- and within-patient estimators under different scenarios**

| **Scenario** | $\boldsymbol{E}\left( {\hat{\boldsymbol{\beta}}}_{\boldsymbol{B}_{\boldsymbol{1}}} \right)$ | $\boldsymbol{E}\left( {\hat{\boldsymbol{\beta}}}_{\boldsymbol{B}_{\boldsymbol{2}}} \right)$ | $\boldsymbol{E}\left( {\hat{\boldsymbol{\beta}}}_{\boldsymbol{W}} \right)$ |
| --- | --- | --- | --- |
| S1 – Constant treatment effect | $\beta$ | $\beta$ | $\beta$ |
| S2 – Treatment effect varies across episode | $\beta_{1}$ | $\frac{1}{2}\left( \beta_{1}+\beta_{2} \right)$ | $\frac{1}{2}\left( \beta_{1}+\beta_{2} \right)$ |
| S3 – Treatment effect varies across value of $M_{i}$ | $\beta_{1}$ | $\beta_{2}$ | $\beta_{2}$ |
| S4 – Treatment effect carries forward into second episode | $\beta$ | $\beta+\frac{\gamma}{2}$ | $\beta-\frac{\gamma}{2}$ |
| S5 – Treatment becomes less effective on re-use | $\beta$ | $\beta+\frac{\delta}{2}$ | $\beta$ |
| S6 – Constant treatment effect, differential non-enrolment based on outcome in previous episode | $\beta_{trt}+\beta_{X_{PL}}\frac{\left( p_{01}-p_{00} \right)}{2\left( 1-p \right)}$ | $\beta_{trt}+\beta_{X_{PL}}\frac{\left( p_{00}-p_{01} \right)}{2p}$ | $\beta_{trt}$ |
| S7 – Constant treatment effect, differential non-enrolment based on expected outcome in current episode | $\beta_{trt}$ | $\beta_{trt}+\frac{\beta_{X_{EL}}}{4}\left( \frac{p_{00}-p_{01}}{p} \right)$ | $\beta_{trt}-\frac{\beta_{X_{EL}}}{4}\left( \frac{p_{00}-p_{01}}{p} \right)$ |

***Scenario 1 (S1): constant treatment effect***

Consider the following data-generating mechanism:

$$Y_{ij}=\alpha+\beta Z_{ij}+\mu_{i}+\varepsilon_{ij} (2)$$

The values of $E\left( Y_{ij} \right)$ for each treatment sequence are shown in Table 7. From this we can see that $E\left( \hat{\beta}_{B_{1}} \right)=E\left( \hat{\beta}_{B_{2}} \right)=E\left( \hat{\beta}_{W} \right)=\beta$.

Therefore:

$$E\left( \hat{\beta}_{E}^{AB} \right)=\frac{\left( 1-p \right)\beta+p\beta+p\beta}{1+p}=\beta$$

And:

$$E\left( \hat{\beta}_{P}^{AB} \right)=\left( 1-p \right)\beta+\frac{p}{2}\beta+\frac{p}{2}\beta=\beta$$

**Table 7 - Value of** $\boldsymbol{E}\left( \boldsymbol{Y}_{\boldsymbol{ij}} \right)$ **across each episode and treatment sequence under *S1: constant treatment effect***

| **Treatment allocation** | | $\boldsymbol{E}\left( \boldsymbol{Y}_{\boldsymbol{ij}} \right)$ | |
| --- | --- | --- | --- |
| Episode 1 | Episode 2 | Episode 1 | Episode 2 |
| 0 | $-$ | 0 | $-$ |
| 0 | 1 | 0 | $\beta$ |
| 0 | 0 | 0 | 0 |
| 1 | $-$ | $\beta$ | $-$ |
| 1 | 0 | $\beta$ | 0 |
| 1 | 1 | $\beta$ | $\beta$ |

***S2: Treatment effect varies across episode***

Consider the following data-generating mechanism:

$$Y_{ij}=\left\{ \begin{aligned} \alpha+\beta_{1}Z_{ij}+\mu_{i}+\varepsilon_{ij}\mathrm{for}j=1 \\ \alpha+\beta_{2}Z_{ij}+\mu_{i}+\varepsilon_{ij}\text{ for }j=2 \end{aligned} \right.$$

The values of $E\left( Y_{ij} \right)$ for each treatment sequence are shown in Table 8. Then, plugging the values from Table 8 into the formulas for the between- and within-patient estimation components, the expected values of the components, $\hat{\beta}_{B_{1}}$, $\hat{\beta}_{B_{2}}$, and $\hat{\beta}_{W}$ are:

$$E\left( \hat{\beta}_{B_{1}} \right)=\beta_{1}$$

$$E\left( \hat{\beta}_{B_{2}} \right)=\frac{1}{2}\left( \beta_{1}+\beta_{2} \right)$$

$$E\left( \hat{\beta}_{W} \right)=\frac{1}{2}\left( \beta_{1}+\beta_{2} \right)$$

Therefore:

$$E\left( \hat{\beta}_{E}^{AB} \right)=\frac{\left( 1-p \right)\beta_{1}+\frac{p}{2}\left( \beta_{1}+\beta_{2} \right)+\frac{p}{2}\left( \beta_{1}+\beta_{2} \right)}{1+p}=\frac{\beta_{1}+p\beta_{2}}{1+p}$$

And:

$$E\left( \hat{\beta}_{P}^{AB} \right)=\left( 1-p \right)\beta_{1}+\frac{p}{2}\frac{\left( \beta_{1}+\beta_{2} \right)}{2}+\frac{p}{2}\frac{\left( \beta_{1}+\beta_{2} \right)}{2}=\left( 1-p+\frac{p}{2} \right)\beta_{1}+\frac{p}{2}\beta_{2}$$

$$=\left( 1-\frac{p}{2} \right)\beta_{1}+\frac{p}{2}\beta_{2}$$

**Table 8 - Value of** $\boldsymbol{E}\left( \boldsymbol{Y}_{\boldsymbol{ij}} \right)$ **across each episode and treatment sequence under *S2: Treatment effect varies across episode***

| **Treatment allocation** | | $\boldsymbol{E}\left( \boldsymbol{Y}_{\boldsymbol{ij}} \right)$ | |
| --- | --- | --- | --- |
| Episode 1 | Episode 2 | Episode 1 | Episode 2 |
| 0 | $-$ | 0 | $-$ |
| 0 | 1 | 0 | $\beta_{2}$ |
| 0 | 0 | 0 | 0 |
| 1 | $-$ | $\beta_{1}$ | $-$ |
| 1 | 0 | $\beta_{1}$ | 0 |
| 1 | 1 | $\beta_{1}$ | $\beta_{2}$ |

***S3: Treatment effect varies across value of*** $\boldsymbol{M}_{\boldsymbol{i}}$

Consider the following data-generating mechanism:

$$Y_{ij}=\left\{ \begin{aligned} \alpha+\beta_{1}Z_{ij}+\mu_{i}+\varepsilon_{ij}\mathrm{if}M_{i}=1 \\ \alpha+\beta_{2}Z_{ij}+\mu_{i}+\varepsilon_{ij}\text{ if }M_{i}=2 \end{aligned} \right.$$

The values of $E\left( Y_{ij} \right)$ for each treatment sequence are shown in Table 9. Then, plugging the values from Table 9 into the formulas for the between- and within-patient estimation components, we can see that the expected values of the components, $\hat{\beta}_{B_{1}}$, $\hat{\beta}_{B_{2}}$, and $\hat{\beta}_{W}$ are:

$$E\left( \hat{\beta}_{B_{1}} \right)=\beta_{1}$$

$$E\left( \hat{\beta}_{B_{2}} \right)=\beta_{2}$$

$$E\left( \hat{\beta}_{W} \right)=\beta_{2}$$

Therefore:

$$E\left( \hat{\beta}_{E}^{AB} \right)=\frac{\left( 1-p \right)\beta_{1}+p\beta_{2}+p\beta_{2}}{1+p}=\frac{\left( 1-p \right)\beta_{1}+2p\beta_{2}}{1+p}$$

And:

$$E\left( \hat{\beta}_{P}^{AB} \right)=\left( 1-p \right)\beta_{1}+\frac{p}{2}\beta_{2}+\frac{p}{2}\beta_{2}=\left( 1-p \right)\beta_{1}+p\beta_{2}$$

**Table 9 - Value of** $\boldsymbol{E}\left( \boldsymbol{Y}_{\boldsymbol{ij}} \right)$ **across each episode and treatment sequence under *S3: Treatment effect varies across value of*** $\boldsymbol{M}_{\boldsymbol{i}}$

| **Treatment allocation** | | $\boldsymbol{E}\left( \boldsymbol{Y}_{\boldsymbol{ij}} \right)$ | |
| --- | --- | --- | --- |
| Episode 1 | Episode 2 | Episode 1 | Episode 2 |
| 0 | $-$ | 0 | $-$ |
| 0 | 1 | 0 | $\beta_{2}$ |
| 0 | 0 | 0 | 0 |
| 1 | $-$ | $\beta_{2}$ | $-$ |
| 1 | 0 | $\beta_{2}$ | 0 |
| 1 | 1 | $\beta_{2}$ | $\beta_{2}$ |

***S4: Treatment effect carries forward into the second episode***

Consider the following data-generating mechanism:

$$Y_{ij}=\alpha+\beta Z_{ij}+\gamma Z_{i,j-1}+\mu_{i}+\varepsilon_{ij}$$

The values of $E\left( Y_{ij} \right)$ for each treatment sequence are shown in Table 10. Then, plugging the values from Table 10 into the formulas for the between- and within-patient estimation components, we can see that the expected values of the components, $\hat{\beta}_{B_{1}}$, $\hat{\beta}_{B_{2}}$, and $\hat{\beta}_{W}$ are:

$$E\left( \hat{\beta}_{B_{1}} \right)=\beta$$

$$E\left( \hat{\beta}_{B_{2}} \right)=\beta+\frac{\gamma}{2}$$

$$E\left( \hat{\beta}_{W} \right)=\beta-\frac{\gamma}{2}$$

Therefore:

$$E\left( \hat{\beta}_{E}^{AB} \right)=\frac{\left( 1-p \right)\beta+p\left( \beta+\frac{\gamma}{2} \right)+p\left( \beta-\frac{\gamma}{2} \right)}{1+p}=\beta$$

And:

$$E\left( \hat{\beta}_{P}^{AB} \right)=\left( 1-p \right)\beta+\frac{p}{2}\left( \beta+\frac{\gamma}{2} \right)+\frac{p}{2}\left( \beta-\frac{\gamma}{2} \right)=\beta$$

**Table 10 - Value of** $\boldsymbol{E}\left( \boldsymbol{Y}_{\boldsymbol{ij}} \right)$ **across each episode and treatment sequence under *S4: Treatment effect carries forward into second episode***

| **Treatment allocation** | | $\boldsymbol{E}\left( \boldsymbol{Y}_{\boldsymbol{ij}} \right)$ | |
| --- | --- | --- | --- |
| Episode 1 | Episode 2 | Episode 1 | Episode 2 |
| 0 | $-$ | 0 | $-$ |
| 0 | 1 | 0 | $\beta$ |
| 0 | 0 | 0 | 0 |
| 1 | $-$ | $\beta$ | $-$ |
| 1 | 0 | $\beta$ | $\gamma$ |
| 1 | 1 | $\beta$ | $\beta+ \gamma$ |

***S5: Treatment becomes less effective on re-use***

Consider the following data-generating mechanism:

$$Y_{ij}=\left\{ \begin{aligned} \alpha+\beta Z_{ij}+\mu_{i}+\varepsilon_{ij}\text{ for }Z_{i,j-1}=0 \\ \alpha+\left( \beta+\delta\right)Z_{ij}+\mu_{i}+\varepsilon_{ij}\text{ for }Z_{i,j-1}=1 \end{aligned} \right.$$

The values of $E\left( Y_{ij} \right)$ for each treatment sequence are shown in Table 11. Then, plugging the values from Table 11 into the formulas for the between- and within-patient estimation components, we can see that the expected values of the components, $\hat{\beta}_{B_{1}}$, $\hat{\beta}_{B_{2}}$, and $\hat{\beta}_{W}$ are:

$$E\left( \hat{\beta}_{B_{1}} \right)=\beta$$

$$E\left( \hat{\beta}_{B_{2}} \right)=\beta+\frac{\delta}{2}$$

$$E\left( \hat{\beta}_{W} \right)=\beta$$

Therefore:

$$E\left( \hat{\beta}_{E}^{AB} \right)=\frac{\left( 1-p \right)\beta+p\left( \beta+\frac{\delta}{2} \right)+p\beta}{1+p}=\frac{\left( 1+p \right)\beta+\frac{p\delta}{2}}{1+p}=\beta+\frac{p\delta}{2\left( 1+p \right)}$$

And:

$$E\left( \hat{\beta}_{P}^{AB} \right)=\left( 1-p \right)\beta+\frac{p}{2}\left( \beta+\frac{\delta}{2} \right)+\frac{p}{2}\beta=\beta+\frac{p\delta}{4}$$

**Table 11 - Value of** $\boldsymbol{E}\left( \boldsymbol{Y}_{\boldsymbol{ij}} \right)$ **across each episode and treatment sequence under *S5: Treatment becomes less effective on re-use***

| **Treatment allocation** | | $\boldsymbol{E}\left( \boldsymbol{Y}_{\boldsymbol{ij}} \right)$ | |
| --- | --- | --- | --- |
| Episode 1 | Episode 2 | Episode 1 | Episode 2 |
| 0 | $-$ | 0 | - |
| 0 | 1 | 0 | $\beta$ |
| 0 | 0 | 0 | 0 |
| 1 | $-$ | $\beta$ | $-$ |
| 1 | 0 | $\beta$ | 0 |
| 1 | 1 | $\beta$ | $\beta+ \delta$ |

***S6: Constant treatment effect, differential non-enrolment based on outcome in previous episode***

In this section (and the next section) we no longer assume that there is no non-enrolment. Instead, we assume that some patients do not re-enrol for their second episode. In this scenario we consider a situation where non-enrolment is differential between treatment arms depending on their outcome in the first episode; patients who received the intervention in episode 1 and had a good outcome have the same probability of re-enrolling as patients who received control in episode 1 and had a bad outcome, and vice versa.

Consider the following data generating mechanism:

$$Y_{ij}=\alpha+\beta_{trt}Z_{ij}+\beta_{X_{PL}}X_{PL_{i}}+\mu_{i}+\varepsilon_{ij} (3)$$

where $X_{PL_{i}}$ is an unobserved binary patient-level variable (i.e. it is constant across episodes). We use the subscript $PL$ to denote ‘patient-level’. This data generating mechanism is equivalent to model (2), but with the addition of $X_{EL_{ij}}$ (and $\beta_{trt}$ in place of $\beta$), and so the treatment effect is constant across patients and episodes.

In this scenario we assume that high values of $Y_{ij}$ are good, which means that patients for whom $X_{PL_{i}}=1$ have better outcomes if $\beta_{X_{PL}}$ is positive. The purpose of $X_{PL_{i}}$ in this scenario is to allow the non-enrolment to be differential based on the episode 1 outcome; this will be explained further below.

Let $R_{i2}=1$ indicate that patient $i$ is enrolled in the trial for episode 2, and $R_{i2}=0$ denoting non-enrolment for the second episode. Note that $R_{i2}=0$ if $M_{i}=1$.

In this scenario, the probability of re-enrolment depends on two factors: treatment allocation in episode 1 ($Z_{i1}$) and the value of $X_{PL_{i}}$. We use $X_{PL_{i}}$ as a marker of the patient’s outcome in episode 1; for instance, if $\beta_{X_{PL}}$ is positive and if patients with $X_{PL_{i}}=1$ are more likely to re-enrol for episode 2, this indicates that patients with better outcomes in episode 1 are more likely to re-enrol in the trial for their 2nd episode. Incorporating $X_{PL_{i}}$ into data generating model (3) and into the model for the probability of re-enrolment allows the non-enrolment to be differential between treatment arms.

Let $\pi=P\left( X_{PL_{i}}=1 \right)$ (i.e. $\pi$ denotes the probability that $X_{PL_{i}}=1$ for patient $i$), and let $p_{zx}=P\left( R_{i2}=1 | Z_{i1}=z_{i1},X_{PL_{i}}=x_{PL_{i}} \right)$ (i.e. $p_{zx}$ denotes the probability of being re-enrolled for a second episode given $Z_{i1}$ and $X_{PL_{i}}$). So, for example, $p_{00}=P\left( R_{i2}=1 | Z_{i1}=0,X_{PL_{i}}=0 \right)$, and $p_{01}=P\left( R_{i2}=1 | Z_{i1}=0,X_{PL_{i}}=1 \right)$.

For simplicity, we will assume that $\pi=0.5$, and that $p_{00}=p_{11}$ and $p_{01}=p_{10}$. This has two main implications. The first is that an equal number of patients from both episode 1 treatment arms will re-enrol for a second episode (i.e. $P\left( R_{i2}=1 | Z_{i1}=0 \right)=P\left( R_{i2}=1 | Z_{i1}=1 \right)$). Second, different types of patients will re-enrol from each episode 1 treatment group; for example, sicker patients in the episode 1 control arm and healthier patients in the episode 1 intervention arm have the same probability of re-enrolling for a 2nd episode, and vice versa.

Note that in this scenario the following (asymptotic) relationship holds for $p$ (where $p$ represents the proportion of patients enrolled in the trial for two episodes): $p=P\left( R_{i2}=1 | Z_{i1}=0 \right)=P\left( R_{i2}=1 | Z_{i1}=1 \right)=\frac{\left( p_{00} \right)\left( 1-\pi\right)+p_{01}\pi}{\left( 1-\pi\right)+\pi}=p_{00}-\pi p_{00}+\pi p_{01}=\frac{1}{2}\left( p_{00}+p_{01} \right)$. This will be used below.

The number of episodes and expected outcomes for each combination of treatment sequence and $X_{PL_{i}}$ value can be seen in Table 12. For simplicity, we have substituted $p_{10}$ and $p_{11}$ with $p_{01}$ and $p_{00}$ respectively, as these are equal in this scenario.

**Table 12 - Number of observations and expected values in each combination of treatment sequence and value of** $\boldsymbol{X}_{\boldsymbol{P}\boldsymbol{L}_{\boldsymbol{i}}}$ **in *S6: Constant treatment effect, differential non-enrolment based on outcome in previous episode.* E1=episode 1, E2=episode 2**

| $\boldsymbol{X}_{\boldsymbol{P}\boldsymbol{L}_{\boldsymbol{i}}}$ | **Treatment allocation** | | **Number of observations** | | $\boldsymbol{E}\left( \boldsymbol{Y}_{\boldsymbol{ij}} \right)$ | |
| --- | --- | --- | --- | --- | --- | --- |
|  | E1 | E2 | E1 | E2 | E1 | E2 |
| 0 | 0 | $-$ | $\frac{N_{T}}{4}\left( 1-p_{00} \right)$ | $-$ | 0 | $-$ |
| 1 | 0 | $-$ | $\frac{N_{T}}{4}\left( 1-p_{01} \right)$ | $-$ | $\beta_{X_{PL}}$ | $-$ |
| 0 | 0 | 1 | $\frac{N_{T}}{8}p_{00}$ | $\frac{N_{T}}{8}p_{00}$ | 0 | $\beta_{trt}$ |
| 1 | 0 | 1 | $\frac{N_{T}}{8}p_{01}$ | $\frac{N_{T}}{8}p_{01}$ | $\beta_{X_{PL}}$ | $\beta_{trt}+\beta_{X_{PL}}$ |
| 0 | 0 | 0 | $\frac{N_{T}}{8}p_{00}$ | $\frac{N_{T}}{8}p_{00}$ | 0 | 0 |
| 1 | 0 | 0 | $\frac{N_{T}}{8}p_{01}$ | $\frac{N_{T}}{8}p_{01}$ | $\beta_{X_{PL}}$ | $\beta_{X_{PL}}$ |
| 0 | 1 | $-$ | $\frac{N_{T}}{4}\left( 1-p_{00} \right)$ | $-$ | $\beta_{trt}$ | $-$ |
| 1 | 1 | $-$ | $\frac{N_{T}}{4}\left( 1-p_{01} \right)$ | $-$ | $\beta_{trt}+\beta_{X_{PL}}$ | $-$ |
| 0 | 1 | 0 | $\frac{N_{T}}{8}p_{00}$ | $\frac{N_{T}}{8}p_{00}$ | $\beta_{trt}$ | 0 |
| 1 | 1 | 0 | $\frac{N_{T}}{8}p_{01}$ | $\frac{N_{T}}{8}p_{01}$ | $\beta_{trt}+\beta_{X_{PL}}$ | $\beta_{X_{PL}}$ |
| 0 | 1 | 1 | $\frac{N_{T}}{8}p_{00}$ | $\frac{N_{T}}{8}p_{00}$ | $\beta_{trt}$ | $\beta_{trt}$ |
| 1 | 1 | 1 | $\frac{N_{T}}{8}p_{01}$ | $\frac{N_{T}}{8}p_{01}$ | $\beta_{trt}+\beta_{X_{PL}}$ | $\beta_{trt}+\beta_{X_{PL}}$ |

Now, collapsing over $X_{PL_{i}}$ in Table 12 leads to Table 13.

**Table 13 - Number of observations and expected values in each treatment sequence in *S6: Constant treatment effect, differential non-enrolment based on outcome in previous episode*. E1=episode 1, E2=episode 2**

| **Treatment allocation** | | **Number of observations** | | $\boldsymbol{E}\left( \boldsymbol{Y}_{\boldsymbol{ij}} \right)$ | |
| --- | --- | --- | --- | --- | --- |
| E1 | E2 | E1 | E2 | E1 | E2 |
| 0 | $-$ | $\frac{N_{T}}{2}\left( 1-p \right)$ | $-$ | $\beta_{X_{PL}}\frac{\left( 1-p_{01} \right)}{2\left( 1-p \right)}$ | $-$ |
| 0 | 1 | $\frac{N_{T}}{4}p$ | $\frac{N_{T}}{4}p$ | $\beta_{X_{PL}}\frac{p_{01}}{2p}$ | $\beta_{trt}+\beta_{X_{PL}}\frac{p_{01}}{2p}$ |
| 0 | 0 | $\frac{N_{T}}{4}p$ | $\frac{N_{T}}{4}p$ | $\beta_{X_{PL}}\frac{p_{01}}{2p}$ | $\beta_{X_{PL}}\frac{p_{01}}{2p}$ |
| 1 | $-$ | $\frac{N_{T}}{2}\left( 1-p \right)$ | $-$ | $\beta_{trt}+\beta_{X_{PL}}\frac{\left( 1-p_{00} \right)}{2\left( 1-p \right)}$ | $-$ |
| 1 | 0 | $\frac{N_{T}}{4}p$ | $\frac{N_{T}}{4}p$ | $\beta_{trt}+\beta_{X_{PL}}\frac{p_{00}}{2p}$ | $\beta_{X_{PL}}\frac{p_{00}}{2p}$ |
| 1 | 1 | $\frac{N_{T}}{4}p$ | $\frac{N_{T}}{4}p$ | $\beta_{trt}+\beta_{X_{PL}}\frac{p_{00}}{2p}$ | $\beta_{trt}+\beta_{X_{PL}}\frac{p_{00}}{2p}$ |

Then, plugging the values from Table 13 into the formulas for the between- and within-patient estimation components, we obtain:

$$E\left( \hat{\beta}_{B_{1}} \right)=\frac{\frac{N_{T}}{2}\left( 1-p \right)}{\frac{N_{T}}{2}\left( 1-p \right)}\left( \beta_{trt}+\beta_{X_{PL}}\frac{\left( 1-p_{00} \right)}{2\left( 1-p \right)}-\beta_{X_{PL}}\frac{\left( 1-p_{01} \right)}{2\left( 1-p \right)} \right)=\beta_{trt}+\beta_{X_{PL}}\frac{\left( p_{01}-p_{00} \right)}{2\left( 1-p \right)}$$

And:

$$E\left( \hat{\beta}_{B_{2}} \right)=\frac{1}{\frac{N_{T}p}{2}}\left( \frac{2N_{T}p}{4}\left( \beta_{trt}+\beta_{X_{PL}}\frac{p_{00}}{2p} \right)-\frac{2N_{T}p}{4}\left( \beta_{X_{PL}}\frac{p_{01}}{2p} \right) \right)=\beta_{trt}+\beta_{X_{PL}}\frac{\left( p_{00}-p_{01} \right)}{2p}$$

And:

$$E\left( \hat{\beta}_{w} \right)=\frac{1}{\frac{N_{T}p}{2}}\left( \frac{N_{T}p}{4}\left( \beta_{trt}+\beta_{X_{PL}}\frac{p_{00}}{2p}-\beta_{X_{PL}}\frac{p_{00}}{2p} \right)-\frac{N_{T}p}{4}\left( \beta_{X_{PL}}\frac{p_{01}}{2p}-\beta_{trt}-\beta_{X_{PL}}\frac{p_{01}}{2p} \right) \right)=\beta_{trt}$$

Therefore:

$$E\left( \hat{\beta}_{E}^{AB} \right)=\frac{\left( 1-p \right)\left( \beta_{trt}+\beta_{X_{PL}}\frac{\left( p_{01}-p_{00} \right)}{2\left( 1-p \right)} \right)+p\left( \beta_{trt}+\beta_{X_{PL}}\frac{\left( p_{00}-p_{01} \right)}{2p} \right)+p\beta_{trt}}{1+p}=\frac{\beta_{trt}\left( \left( 1-p \right)+p+p \right)+\beta_{X_{PL}}\left( \frac{\left( 1-p \right)\left( p_{01}-p_{00} \right)}{2\left( 1-p \right)}+\frac{p\left( p_{00}-p_{01} \right)}{2p} \right)}{1+p}=\frac{\beta_{trt}\left( 1+p \right)+\frac{\beta_{X_{PL}}}{2}\left( p_{01}-p_{00}+p_{00}-p_{01} \right)}{1+p}=\beta_{trt}$$

And:

$$E\left( \hat{\beta}_{P}^{AB} \right)=\left( 1-p \right)\left( \beta_{trt}+\beta_{X_{PL}}\frac{\left( p_{01}-p_{00} \right)}{2\left( 1-p \right)} \right)+\frac{p}{2}\left( \beta_{trt}+\beta_{X_{PL}}\frac{\left( p_{00}-p_{01} \right)}{2p} \right)+\frac{p}{2}\beta_{trt}=\beta_{trt}\left( 1-p+\frac{p}{2}+\frac{p}{2} \right)+\beta_{X_{PL}}\left( \left( 1-p \right)\frac{\left( p_{01}-p_{00} \right)}{2\left( 1-p \right)}+\frac{p}{2}\frac{\left( p_{00}-p_{01} \right)}{2p} \right)=\beta_{trt}+\beta_{X_{PL}}\frac{\left( p_{01}-p_{00} \right)}{4}$$

***S7: Constant treatment effect, differential non-enrolment based on expected outcome in current episode***

In this scenario we consider a situation where non-enrolment is differential between treatment arms depending on their expected outcome in the second episode (i.e. their baseline prognosis at episode 2); patients who received the intervention in episode 1 and have a good baseline prognosis at episode 2 have the same probability of re-enrolling as patients who received control in episode 1 and had a bad prognosis, and vice versa.

Consider the following data generating mechanism:

$$Y_{ij}=\alpha+\beta_{trt}Z_{ij}+\beta_{X_{EL}}X_{EL_{ij}}+\mu_{i}+\varepsilon_{ij}$$

where $X_{EL_{ij}}$ is an unobserved binary episode-level variable (i.e. it can vary across episodes). We use the subscript $EL$ to denote ‘episode-level’. This data generating mechanism is equivalent to model (2), but with the addition of $X_{EL_{ij}}$ (and $\beta_{trt}$ in place of $\beta$), and so the treatment effect is constant across patients and episodes.

As in the previous scenario, high values of $Y_{ij}$ are good, which means that patients for whom $X_{EL_{ij}}=1$ have better outcomes in episode $j$ if $\beta_{X_{EL}}$ is positive. The purpose of $X_{EL_{ij}}$ in this scenario is to allow the non-enrolment to be differential based on the expected outcome at episode 2 (i.e. based on the baseline prognosis at episode 2); this will be explained further below.

In this scenario, we will redefine $\pi$ as $\pi=P\left( X_{EL_{ij}}=1 \right)$, and set this to $\pi=0.5$. Similarly we will redefine $p_{zx}$ as $p_{zx}=P\left( R_{i2}=1 | Z_{i1}=z_{i1},X_{EL_{i2}}=x_{EL_{i2}} \right)$ (so $p_{zx}$ represents the probability of re-enrolment for a second episode based on the patient’s episode 1 allocation and their episode 2 value of $X_{EL_{ij}}$). So, for example, $p_{00}=P\left( R_{i2}=1 | Z_{i1}=0,X_{EL_{i2}}=0 \right)$, and $p_{01}=P\left( R_{i2}=1 | Z_{i1}=0,X_{EL_{i2}}=1 \right)$.

As before, we will assume that $p_{00}=p_{11}$ and $p_{01}=p_{10}$. This, combined with setting $\pi=0.5$ above, implies that an equal number of patients from both episode 1 treatment arms will re-enrol for a second episode (i.e. $P\left( R_{i2}=1 | Z_{i1}=0 \right)=P\left( R_{i2}=1 | Z_{i1}=1 \right)$), and that different types of patients will re-enrol from each episode 1 treatment group (for example, patients with a worse prognosis at episode 2 who received control in episode 1 have the same probability of re-enrolling as patients with a better prognosis at episode 2 who received intervention in episode 1, and vice versa).

As in the previous scenario, note that $p=P\left( R_{i2}=1 | Z_{i1}=0 \right)=P\left( R_{i2}=1 | Z_{i1}=1 \right)=\frac{\left( p_{00} \right)\left( 1-\pi\right)+p_{01}\pi}{\left( 1-\pi\right)+\pi}=p_{00}-\pi p_{00}+\pi p_{01}=\frac{1}{2}\left( p_{00}+p_{01} \right)$.

In this scenario, we will assume for simplicity that all patients experienced two episodes (i.e. $M_{i}=2$ for all patients), but that some of these patients were not re-enrolled for their 2nd episode. Therefore, even patients who were enrolled for only a single episode still have a value for $X_{EL_{i2}}$.

The number of episodes and expected outcomes for each sequence can be seen in Table 14; note that for simplicity we have replaced $p_{10}$ and $p_{11}$ with $p_{01}$ and $p_{00}$ respectively, as these are equal in this scenario.

**Table 14 - Number of observations and expected values in each combination of treatment sequence and value of** $\boldsymbol{X}_{\boldsymbol{E}\boldsymbol{L}_{\boldsymbol{i}}}$ **in *S7: Constant treatment effect, differential non-enrolment based on expected outcome in current episode*. E1=episode 1, E2=episode 2**

| $\boldsymbol{X}_{\boldsymbol{E}\boldsymbol{L}_{\boldsymbol{i}}}$ | | **Treatment allocation** | | **Number of observations** | | $\boldsymbol{E}\left( \boldsymbol{Y}_{\boldsymbol{ij}} \right)$ | |
| --- | --- | --- | --- | --- | --- | --- | --- |
| E1 | E2 | E1 | E2 | E1 | E2 | E1 | E2 |
| 0 | 0 | 0 | $-$ | $\frac{N_{T}}{8}\left( 1-p_{00} \right)$ | $-$ | 0 | $-$ |
| 0 | 1 | 0 | $-$ | $\frac{N_{T}}{8}\left( 1-p_{01} \right)$ | $-$ | 0 | $-$ |
| 1 | 0 | 0 | $-$ | $\frac{N_{T}}{8}\left( 1-p_{00} \right)$ | $-$ | $\beta_{X_{EL}}$ | $-$ |
| 1 | 1 | 0 | $-$ | $\frac{N_{T}}{8}\left( 1-p_{01} \right)$ | $-$ | $\beta_{X_{EL}}$ | $-$ |
| 0 | 0 | 0 | 1 | $\frac{N_{T}}{16}p_{00}$ | $\frac{N_{T}}{16}p_{00}$ | 0 | $\beta_{trt}$ |
| 0 | 1 | 0 | 1 | $\frac{N_{T}}{16}p_{01}$ | $\frac{N_{T}}{16}p_{01}$ | 0 | $\beta_{trt}+\beta_{X_{EL}}$ |
| 1 | 0 | 0 | 1 | $\frac{N_{T}}{16}p_{00}$ | $\frac{N_{T}}{16}p_{00}$ | $\beta_{X_{EL}}$ | $\beta_{trt}$ |
| 1 | 1 | 0 | 1 | $\frac{N_{T}}{16}p_{01}$ | $\frac{N_{T}}{16}p_{01}$ | $\beta_{X_{EL}}$ | $\beta_{trt}+\beta_{X_{EL}}$ |
| 0 | 0 | 0 | 0 | $\frac{N_{T}}{16}p_{00}$ | $\frac{N_{T}}{16}p_{00}$ | 0 | 0 |
| 0 | 1 | 0 | 0 | $\frac{N_{T}}{16}p_{01}$ | $\frac{N_{T}}{16}p_{01}$ | 0 | $\beta_{X_{EL}}$ |
| 1 | 0 | 0 | 0 | $\frac{N_{T}}{16}p_{00}$ | $\frac{N_{T}}{16}p_{00}$ | $\beta_{X_{EL}}$ | 0 |
| 1 | 1 | 0 | 0 | $\frac{N_{T}}{16}p_{01}$ | $\frac{N_{T}}{16}p_{01}$ | $\beta_{X_{EL}}$ | $\beta_{X_{EL}}$ |
| 0 | 0 | 1 | $-$ | $\frac{N_{T}}{8}\left( 1-p_{01} \right)$ | $-$ | $\beta_{trt}$ | $-$ |
| 0 | 1 | 1 | $-$ | $\frac{N_{T}}{8}\left( 1-p_{00} \right)$ | $-$ | $\beta_{trt}$ | $-$ |
| 1 | 0 | 1 | $-$ | $\frac{N_{T}}{8}\left( 1-p_{01} \right)$ | $-$ | $\beta_{trt}+\beta_{X_{EL}}$ | $-$ |
| 1 | 1 | 1 | $-$ | $\frac{N_{T}}{8}\left( 1-p_{00} \right)$ | $-$ | $\beta_{trt}+\beta_{X_{EL}}$ | $-$ |
| 0 | 0 | 1 | 0 | $\frac{N_{T}}{16}p_{01}$ | $\frac{N_{T}}{16}p_{01}$ | $\beta_{trt}$ | 0 |
| 0 | 1 | 1 | 0 | $\frac{N_{T}}{16}p_{00}$ | $\frac{N_{T}}{16}p_{00}$ | $\beta_{trt}$ | $\beta_{X_{EL}}$ |
| 1 | 0 | 1 | 0 | $\frac{N_{T}}{16}p_{01}$ | $\frac{N_{T}}{16}p_{01}$ | $\beta_{trt}+\beta_{X_{EL}}$ | 0 |
| 1 | 1 | 1 | 0 | $\frac{N_{T}}{16}p_{00}$ | $\frac{N_{T}}{16}p_{00}$ | $\beta_{trt}+\beta_{X_{EL}}$ | $\beta_{X_{EL}}$ |
| 0 | 0 | 1 | 1 | $\frac{N_{T}}{16}p_{01}$ | $\frac{N_{T}}{16}p_{01}$ | $\beta_{trt}$ | $\beta_{trt}$ |
| 0 | 1 | 1 | 1 | $\frac{N_{T}}{16}p_{00}$ | $\frac{N_{T}}{16}p_{00}$ | $\beta_{trt}$ | $\beta_{trt}+\beta_{X_{EL}}$ |
| 1 | 0 | 1 | 1 | $\frac{N_{T}}{16}p_{01}$ | $\frac{N_{T}}{16}p_{01}$ | $\beta_{trt}+\beta_{X_{EL}}$ | $\beta_{trt}$ |
| 1 | 1 | 1 | 1 | $\frac{N_{T}}{16}p_{00}$ | $\frac{N_{T}}{16}p_{00}$ | $\beta_{trt}+\beta_{X_{EL}}$ | $\beta_{trt}+\beta_{X_{EL}}$ |

Now, collapsing over $X_{EL_{ij}}$ in Table 14 leads to Table 15 below.

**Table 15 - Number of observations and expected values in each treatment sequence in *S7: Constant treatment effect, differential non-enrolment based on expected outcome in current episode*. E1=episode 1, E2=episode 2**

| **Treatment allocation** | | **Number of observations** | | $\boldsymbol{E}\left( \boldsymbol{Y}_{\boldsymbol{ij}} \right)$ | |
| --- | --- | --- | --- | --- | --- |
| E1 | E2 | E1 | E2 | E1 | E2 |
| 0 | $-$ | $\frac{N_{T}}{2}\left( 1-p \right)$ | $-$ | $\frac{\beta_{X_{EL}}}{2}$ | $-$ |
| 0 | 1 | $\frac{N_{T}}{4}p$ | $\frac{N_{T}}{4}p$ | $\frac{\beta_{X_{EL}}}{2}$ | $\beta_{trt}+\beta_{X_{EL}}\frac{p_{01}}{p_{00}+p_{01}}$ |
| 0 | 0 | $\frac{N_{T}}{4}p$ | $\frac{N_{T}}{4}p$ | $\frac{\beta_{X_{EL}}}{2}$ | $\beta_{X_{EL}}\frac{p_{01}}{p_{00}+p_{01}}$ |
| 1 | $-$ | $\frac{N_{T}}{2}\left( 1-p \right)$ | $-$ | $\beta_{trt}+\frac{\beta_{X_{EL}}}{2}$ | $-$ |
| 1 | 0 | $\frac{N_{T}}{4}p$ | $\frac{N_{T}}{4}p$ | $\beta_{trt}+\frac{\beta_{X_{EL}}}{2}$ | $\beta_{trt}+\beta_{X_{EL}}\frac{p_{00}}{p_{00}+p_{01}}$ |
| 1 | 1 | $\frac{N_{T}}{4}p$ | $\frac{N_{T}}{4}p$ | $\beta_{trt}+\frac{\beta_{X_{EL}}}{2}$ | $\beta_{trt}+\beta_{X_{EL}}\frac{p_{00}}{p_{00}+p_{01}}$ |

Then, plugging the values from Table 15 into the formulas for the between- and within-patient estimation components, we obtain:

$$E\left( \hat{\beta}_{B_{1}} \right)=\frac{\frac{N_{T}}{2}\left( 1-p \right)}{\frac{N_{T}}{2}\left( 1-p \right)}\left( \beta_{trt}+\frac{\beta_{X_{EL}}}{2}-\frac{\beta_{X_{EL}}}{2} \right)=\beta_{trt}$$

And:

$$E\left( \hat{\beta}_{B_{2}} \right)=\frac{\frac{N_{T}p}{4}}{\frac{N_{T}p}{2}}\left( \beta_{trt}+\frac{\beta_{X_{EL}}}{2}+\beta_{trt}+\beta_{X_{EL}}\frac{p_{00}}{p_{00}+p_{01}}-\frac{\beta_{X_{EL}}}{2}-\beta_{X_{EL}}\frac{p_{01}}{p_{00}+p_{01}} \right)$$

$$=\frac{1}{2}\left( 2\beta_{trt}+\beta_{X_{EL}}\left( \frac{p_{00}-p_{01}}{p_{00}+p_{01}} \right) \right)=\beta_{trt}+\frac{\beta_{X_{EL}}}{4}\left( \frac{p_{00}-p_{01}}{p} \right)$$

And:

$$E\left( \hat{\beta}_{w} \right)=\frac{1}{\frac{N_{T}p}{2}}\left( \frac{N_{T}p}{4}\left( \beta_{trt}+\frac{\beta_{X_{EL}}}{2}-\beta_{X_{EL}}\frac{p_{00}}{p_{00}+p_{01}}-\frac{\beta_{X_{EL}}}{2}+\beta_{trt}+\beta_{X_{EL}}\frac{p_{01}}{p_{00}+p_{01}} \right) \right)$$

$$=\frac{1}{2}\left( 2\beta_{trt}-\beta_{X_{EL}}\left( \frac{p_{00}-p_{01}}{p_{00}+p_{01}} \right) \right)=\beta_{trt}-\frac{\beta_{X_{EL}}}{4}\left( \frac{p_{00}-p_{01}}{p} \right)$$

Therefore:

$$E\left( \hat{\beta}_{E}^{AB} \right)=\frac{\left( 1-p \right)\beta_{trt}+p\left( \beta_{trt}+\frac{\beta_{X_{EL}}}{4}\left( \frac{p_{00}-p_{01}}{p} \right) \right)+p\left( \beta_{trt}-\frac{\beta_{X_{EL}}}{4}\left( \frac{p_{00}-p_{01}}{p} \right) \right)}{1+p}=\frac{\left( 1-p+p+p \right)\beta_{trt}+\frac{p\beta_{X_{EL}}}{4}\left( \frac{p_{00}-p_{01}-p_{00}+p_{01}}{p} \right)}{1+p}=\beta_{trt}$$

And:

$$E\left( \hat{\beta}_{P}^{AB} \right)=\left( 1-p \right)\beta_{trt}+\frac{p}{2}\left( \beta_{trt}+\frac{\beta_{X_{EL}}}{4}\left( \frac{p_{00}-p_{01}}{p} \right) \right)+\frac{p}{2}\left( \beta_{trt}-\frac{\beta_{X_{EL}}}{4}\left( \frac{p_{00}-p_{01}}{p} \right) \right)=\left( 1-p+\frac{p}{2}+\frac{p}{2} \right)\beta_{trt}+\frac{p\beta_{X_{EL}}}{8}\left( \frac{p_{00}-p_{01}-p_{00}+p_{01}}{p} \right)=\beta_{trt}$$

**Summary of mathematical results**

A summary of results for the per-episode added-benefit and the per-patient added-benefit estimators are available in Tables 2 and 3. From Table 2, we can see that the per-episode added-benefit estimator is unbiased in all settings considered.

From Table 3, we can see that the per-patient added-benefit estimator is unbiased, except when there is differential non-enrolment based on the episode 1 outcome. The reason for this bias may be that the association between $Y_{ij}$ and $M_{i}$ is different between treatment groups in this scenario. Consider the following example; imagine a trial where there is no difference between treatment groups (i.e. a null treatment effect). However, the association between $Y_{ij}$ and $M_{i}$ differs between groups as follows; good values of $Y_{ij}$ in the control group tend to be associated with episodes where $M_{i}=1$, whereas good values of $Y_{ij}$ in the intervention group tend to be associated with episodes where $M_{i}=2$. Then, when the weights $W_{i}=\frac{1}{M_{i}}$ are applied to outcomes $Y_{ij}$, good values of $Y_{ij}$ in the control group are down weighted less than good values in the intervention group, which would cause a difference between treatment groups even though none exists.

This is what occurred in this scenario. Patients in the intervention group with good outcomes in episode 1 were less likely to re-enrol for episode 2. This meant that episodes with good outcomes in the intervention arm were more likely to have $M_{i}=1$ than in the control group. Likewise, episodes with good outcomes in the control group were more likely to have $M_{i}=2$ than in the intervention group (formally, there was a higher proportion of episodes where $M_{i}=1$ and $X_{PL_{i}}=1$ in the intervention arm than in the control, and a higher proportion where $M_{i}=2$ and $X_{PL_{i}}=0$ in the control arm). This lead to good outcomes being weighted differently between the treatment groups, which caused bias in the estimated treatment effect.

**References**

1. Kahan BC, Forbes AB, Dore CJ, Morris TP. A re-randomisation design for clinical trials. BMC Med Res Methodol. 2015;15:96.
